# DNA polymerases η and κ bypass *N*^2^-guanine-*O*^6^-alkylguanine DNA alkyltransferase cross-linked DNA-peptides

**DOI:** 10.1016/j.jbc.2021.101124

**Published:** 2021-08-28

**Authors:** Pratibha P. Ghodke, F. Peter Guengerich

**Affiliations:** Department of Biochemistry, Vanderbilt University School of Medicine, Nashville, Tennessee, USA

**Keywords:** DNA cross-link, DNA polymerase, DNA enzyme;, DNA damage, DNA–protein interaction, DNA repair, fidelity of DNA synthesis, DNA damage response, DNA–peptide cross-link, DNA–protein cross-link, AGT, *O*^6^-alkylguanine DNA-alkyl transferase, CID, collision-induced dissociation, CPG, controlled pore glass, DBU, 1,8-dizabicyclo[5.4.0]undec-7-ene, dha, dehydroalanine, DMSO, dimethylsulfoxide, DTT, dithiothreitol, dU, deoxyuridine, ESI, electrospray ionization (mass spectrometry), FAM, 6-carboxyfluorescein, 2-F-dI, 2-fluorodeoxyinosine, h, human, LC-ESI-MS/MS, combined liquid chromatography-electrospray ionization-tandem mass spectrometry, MALDI, matrix-assisted laser desorption ionization (mass spectrometry), MS, mass spectrometry, MSH, *O*-(mesitylsulfonyl)hydroxylamine, ONPE, *O*-(*p*-nitrophenylethyl), PAGE, polyacrylamide gel electrophoresis, pol, polymerase, TLS, translesion (DNA) synthesis, TOF, time-of-flight (mass spectrometry), UDG, uracil DNA glycosylase, UPLC, ultraperformance liquid chromatography

## Abstract

DNA-protein cross-links are formed when proteins become covalently trapped with DNA in the presence of exogenous or endogenous alkylating agents. If left unrepaired, they inhibit transcription as well as DNA unwinding during replication and may result in genome instability or even cell death. The DNA repair protein *O*^6^-alkylguanine DNA-alkyltransferase (AGT) is known to form DNA cross-links in the presence of the carcinogen 1,2-dibromoethane, resulting in G:C to T:A transversions and other mutations in both bacterial and mammalian cells. We hypothesized that AGT-DNA cross-links would be processed by nuclear proteases to yield peptides small enough to be bypassed by translesion (TLS) polymerases. Here, a 15-mer and a 36-mer peptide from the active site of AGT were cross-linked to the N2 position of guanine *via* conjugate addition of a thiol containing a peptide dehydroalanine moiety. Bypass studies with DNA polymerases (pols) η and κ indicated that both can accurately bypass the cross-linked DNA peptides. The specificity constant (*k*_cat_/*K*_m_) for steady-state incorporation of the correct nucleotide dCTP increased by 6-fold with human (h) pol κ and 3-fold with hpol η, with hpol η preferentially inserting nucleotides in the order dC > dG > dA > dT. LC-MS/MS analysis of the extension product also revealed error-free bypass of the cross-linked 15-mer peptide by hpol η. We conclude that a bulky 15-mer AGT peptide cross-linked to the N2 position of guanine can retard polymerization, but that overall fidelity is not compromised because only correct bases are inserted and extended.

DNA is continuously being damaged by both endogenous and exogenous agents (1, 2). Such DNA damage affects multiple cellular processes including DNA replication and repair (2). Among the various kinds of DNA damage, the formation of bulky DNA–protein cross-link adducts can have a strong impact on chromatin-based processes and also contribute to the toxicity if left unrepaired (3, 4, 5). DNA–protein cross-links can be formed in many different ways (6): (i) by endogenous agents either in enzymatic (*e.g.*, topoisomerase) (7) or nonenzymatic processes (*e.g.*, abasic sites, formaldehyde) (8, 9); (ii) by chemotherapeutic and other exogenous chemical agents (*e.g.*, cisplatin-induced DNA-protein cross-links) (10); (iii) by exogenous physical damage (*e.g.*, ionizing radiation) (11); or (iv) if a protein strongly binds to DNA and behaves as a DNA–protein cross-link. Proteins involved in cross-linking include DNA polymerase (pol) β (12), poly(ADP) ribose polymerase 1 (PARP1) (13), histones (14), DNA glycosylase (15), HMCES (16), and at least 70 others (17). DNA–protein cross-links can form at various sites in DNA, including all four natural nucleobases (dA, dC, dG, and dT). In addition to all these positions, reported DNA–protein cross-links at modified nucleobases include abasic sites (18), 5-formyl-dC (19), and *N*^7^-Me-dG (20).

The repair of DNA–protein cross-links is more challenging due to its diverse nature, and more remains to be discovered. The repair process involves all three moieties, *i.e.*, protein, DNA, or the attachment between DNA and protein (21, 22). Nuclear proteases and proteasome-based repair target the protein moieties in DNA–protein cross-links and degrade proteins into peptides to reduce their bulkiness and prevent the toxicity generated by these cross-links (23). Proteases involved in repair processes include Wss1, SPRTN, GCNA, and FAM111A and B (24), although only Wss1 and SPRTN have been shown to act directly. Posttranslational modification of proteases *via* binding to ubiquitin or SUMO can play an important role in proteolysis (25). Nuclease-based repair targets the DNA moiety of DNA–protein cross-links (*e.g.*, MRN (Mre11–Rad50–Xrs2) complex in the processing of topoisomerase II cross-links at the ends of double-strand breaks) (26). The third target is the attachment between DNA and protein that can be hydrolyzed to release protein from the DNA (*e.g.*, tyrosyl-DNA phosphodiesterase (TDP), an enzyme capable of breaking down the covalent attachment between topoisomerases and DNA) (27). In addition, DNA–protein cross-links can also be repaired by homologous recombination and nucleotide excision repair pathways (28), although the latter have some limitations (29).

If bulky DNA–protein cross-links are left unrepaired, they can be very detrimental to cells as major obstacles for cellular processes such as replication, DNA repair, recombination, chromatin remodeling, and transcription (3). DNA–protein cross-links are thought to contribute to various mutagenic events, genomic instability, and even cell death, in that defects in protease-mediated DNA–protein cross-link repair are associated with aging and cancer, *e.g.*, Ruijs–Aalfs syndrome linked to mutations in SPRTN (30), pediatric germ cell tumors (31), Kenny–Caffey syndrome type 2 (32), and gracile bone dysplasia (33).

The DNA repair protein *O*^6^-alkylguanine DNA-alkyltransferase (AGT or MGMT) is known to form cross-links with DNA in the presence of bifunctional electrophiles (*i.e.*, 1,2-dibromoethane) resulting in G:C to T:A transversions and other mutations in both *Escherichia coli* and CHO cells (34, 35, 36). The mechanism of formation of AGT-DNA cross-links involves the nucleophilic attack of the active site residue Cys-145 on 1,2-dibromoethane and leads to the formation of a half-mustard intermediate, which further cyclizes into an unstable episulfonium ion (37). Nucleophilic sites on DNA react with the unstable episulfonium ion and form AGT-DNA cross-links (34, 35), including the N6 position of dA, N7 position of dG, N2 position of dG, N1 position of dG, and O6 position of dG (37). This process is related to the induction of mutations in both *E. coli* and mammalian cells (34, 35, 36).

The processing (and possible repair) of AGT-DNA cross-links are not fully understood. Our current hypothesis is that such AGT-DNA cross-links are processed by proteases to yield peptides that are small enough to be bypassed by human translesion (TLS) DNA polymerases, with the introduction of mutations (Fig. 1). The focus of our work was the synthesis and bypass studies with DNA-peptide cross-links from the active site of AGT at the N2 position of dG, known to occur in DNA (37) (Fig. 1). *N*^2^-dG-DNA-peptide cross-links with varying bulk (15- and 36-amino acids) were synthesized using the strategy involving the conjugate addition of a thiol-containing oligonucleotide with a dehydroalanine moiety formed from AGT in the Cys-145 peptides, thus yielding the same basic AGT-DNA cross-link as formed with 1,2-dibromoethane. Our results indicate that both hpol η and hpol κ bypass these long peptides and insert bases correctly across from the *N*^2^-dG-DNA-peptide cross-links.

**Figure 1:**
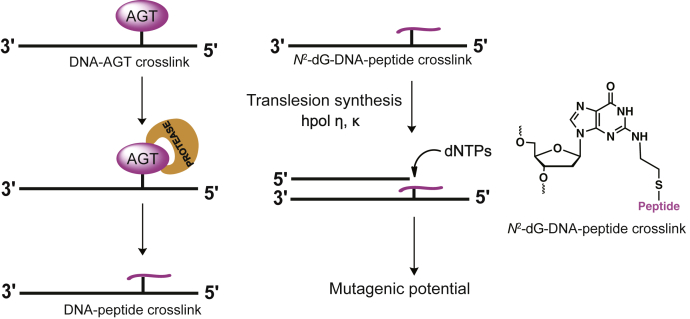
**Proposed processing of 1,2-dibromoethane induced AGT-*N***^**2**^**-dG-DNA cross-link and translesion synthesis across from *N***^**2**^**-dG-oligonucleotide-peptide cross-link**.

## Results

### Synthesis, purification, and characterization of *N*^2^-dG-15 mer and 36-mer peptide oligonucleotide cross-links

The *N*^2^-dG DNA peptide cross-links were synthesized using a 2-fluorodeoxyinosine (2-F-dI)-containing 19-nucleotide oligonucleotide and 15-mer (Ac-PVPILIPCHRVVSSS-NH_2_, AGT residues 138–152, with Cys-150 changed to Ser) or a 36-mer (Ac-PLAARAVGGALRGNPVPILIPCHRVVSSSGAVGNYS-NH_2_, AGT residues 124–159, with Lys-125 and Met-134 both changed to Leu and Cys-150 changed to Ser to yield modification only at the Cys-145 residue) peptide (see Table S1 for oligonucleotide and peptide sequences). The 15-mer (38, 39) and 36-mer peptides were treated with *O*-(mesitylsulfonyl)hydroxylamine (MSH) to convert the cysteine residue to dehydroalanine (dha) (Fig. S1) (40). The respective 15-mer (39) and 36-mer dha-modified peptides were purified by HPLC (Fig. S2) and characterized by positive ion nanoLC-MS (Fig. S3).

**Figure 2:**
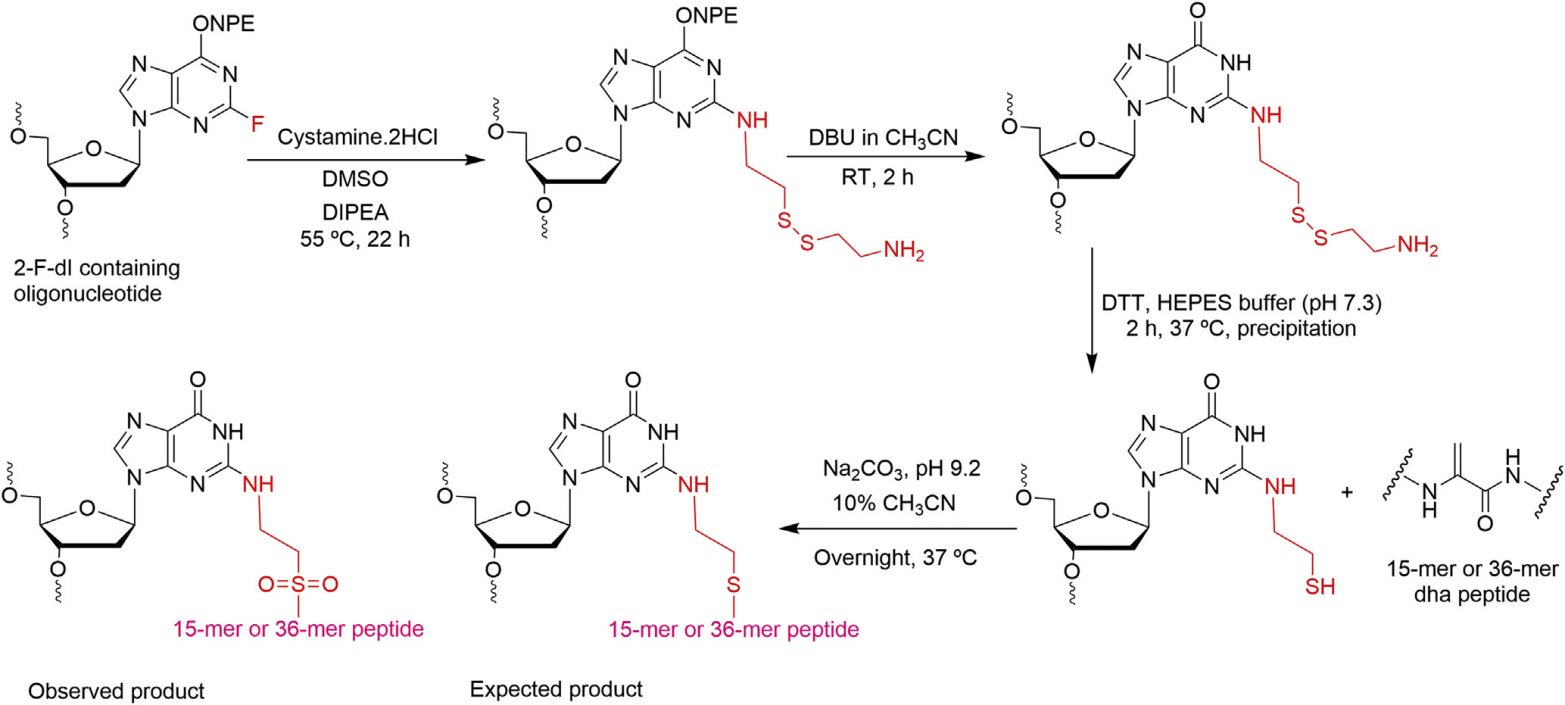
**Synthetic strategy for *N***^**2**^**-dG-15-mer as well as 36-mer peptide DNA cross-links.** DBU, 1,8-dizabicyclo[5.4.0]undec-7-ene; DIPEA, *N*,*N*-diisopropyl,*N*-ethylamine; DMSO, dimethylsulfoxide; ONPE, *O*^6^-(*p*-nitrophenylethyl).

A postoligomerization approach was developed for the synthesis of the oligonucleotide-peptide cross-links. The 2-F-dI-containing oligonucleotide was converted into an *N*^2^-cystamine-dG-modified oligonucleotide (Fig. 2). The synthesis was carried out on a CPG (controlled pore glass) level, which involves nucleophilic displacement using cystamine followed by *O*^6^-(*p*-nitrophenylethyl) (NPE) deprotection (41). The final oligonucleotide deprotection was carried out to cleave CPG beads and to remove base-protecting groups to obtain *N*^2^-cystamine-dG-modified oligonucleotide (Fig. 2). The cystamine-modified oligonucleotide was purified by HPLC (Fig. S4) and characterized by MALDI MS (Fig. S5).

**Figure 3:**
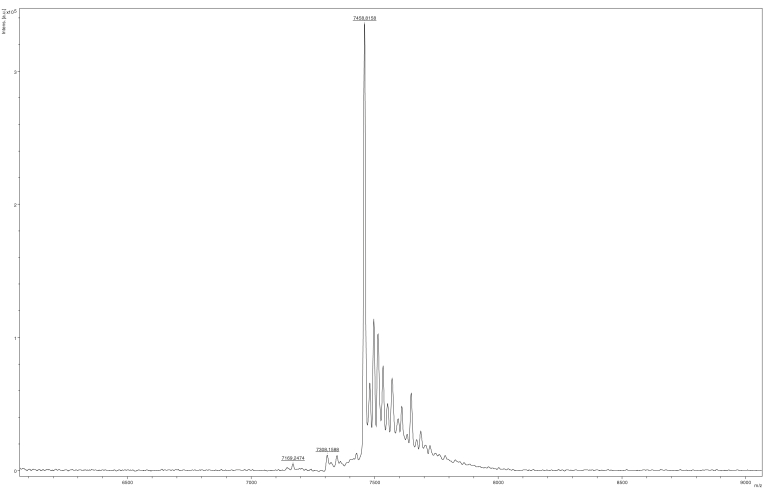
**MALDI mass spectrum of oxidized *N***^**2**^**-dG-15-mer peptide:19-mer oligonucleotide cross-link.** Expected mass [MH]^+^ 7459.8097, observed mass [M + H]^+^ 7458.8158.

The cystamine-containing oligonucleotide was reduced with DTT to obtain an *N*^2^-(2-thioethyl)-dG oligonucleotide (Fig. 2), which was coupled with each respective dha-containing peptide (Fig. 2). The DNA–peptide cross-links were purified by gel electrophoresis (Fig. S6), and the *N*^2^-dG-15-mer peptide cross-link was characterized by MALDI MS (Fig. 3). To simplify the LC-MS/MS characterization, the nucleic acid moiety of the oligonucleotide–peptide cross-links was hydrolyzed with HF to generate a peptide adducted with only the base guanine (Fig. S7) and analyzed using nano-LC-MS/MS in the positive mode (Figs. S8 and S9; Tables S2 and S3). As a result, oxidized sulfur-bearing oligonucleotide–peptide cross-links were observed with an additional mass of 32 a. m. u. (Fig. 2). The major ions at *m/z* 927.56 (+2) (Fig. S8) for the *N*^2^-dG-15-mer-peptide cross-link and at *m/z* 952.29 (+4) (Fig. S9) for the *N*^2^-dG-36-mer-peptide cross-link and their fragmentation patterns (Figs. S8*B* and S9*B*; Tables S2 and S3) indicated an oxidized sulfur atom of the cysteine (sulfone). These oxidized forms of oligonucleotide–peptide cross-links, which had also been observed in our work with *N*^6^-adenyl cross-links to the AGT peptides (39), were used for further studies.

### Human TLS pol η- and pol κ-mediated bypass of *N*^2^-dG-peptide cross-links

Full-length extension and single-nucleotide incorporation assays were carried out with two major human (h) TLS pols, η and κ. Preliminary studies with shorter peptides (3-, 5-, 7-, and 11-mers) bound at the N6 atom of dA, prepared using the same dehydroalanine strategy (39, 40), were bypassed readily by these two polymerases, and we focused our work on the AGT active site 15-mer peptide bound at the N2 atom of dG, in that it was more informative of longer peptides that might be present in cells. In the course of our work, we extended the studies to a 36-mer AGT active site peptide cross-link at the N2 atom of dG, although we did not repeat all of the miscoding studies.

**Figure 4:**
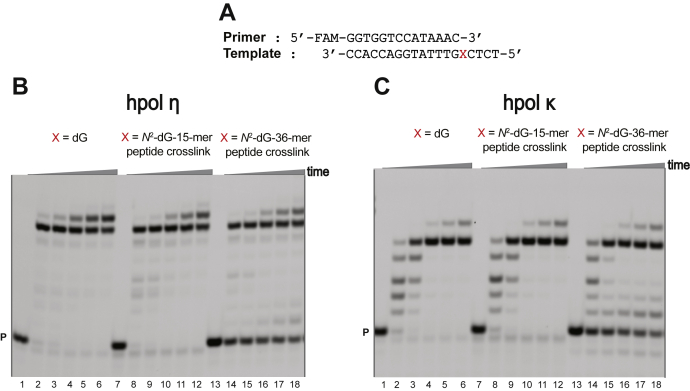
**Full-length extension by hpol η and hpol κ in the presence of all four dNTPs.***A*, 14-mer primer and 19-mer template DNA sequences, where X is dG, *N*^2^-dG-15-mer peptide cross-link, or *N*^2^-dG-15-mer peptide cross-link. Reactions were done in the presence of: *B*, 20 nM hpol η; *C*, 20 nM hpol κ. All reactions were done at 37 °C for 0, 5, 10-, 30-, 60-, or 120-min. Lanes 1–6 have X as dG, lanes 7–12 have X as *N*^2^-dG-15-mer peptide cross-link, and lanes 13–18 have X as *N*^2^-dG-36-mer peptide cross-link. P, FAM-labeled 14-mer DNA primer.

The full-length extension reactions were performed using a 14-mer primer and respective unmodified and cross-linked templates in the presence of mixture of dNTPs (Fig. 4*A*). With the *N*^2^-dG-15-mer peptide cross-link, hpol η fully extended the primer with similar efficiency as the unmodified template (Fig. 4*B*, lanes 1–6 and 7–12). In the case of the *N*^2^-dG-36-mer peptide cross-link, the extension efficiency was reduced as compared with the unmodified as well as the *N*^2^-dG-15-mer peptide cross-link template (Fig. 4*B*, lanes 13–18).

With the *N*^2^-dG-15-mer peptide cross-link, hpol κ also fully extended the primer with similar efficiency as the unmodified template (Fig. 4*C*, lanes 1–6 and 7–12). In the case of the *N*^2^-dG-36-mer peptide cross-link, the extension efficiency was reduced as compared with the unmodified template as well as the *N*^2^-dG-15-mer peptide cross-link template (Fig. 4*C*, lanes 13–18). Overall, both of the TLS polymerases were able to tolerate the 36-amino acid bulk.

**Figure 5:**
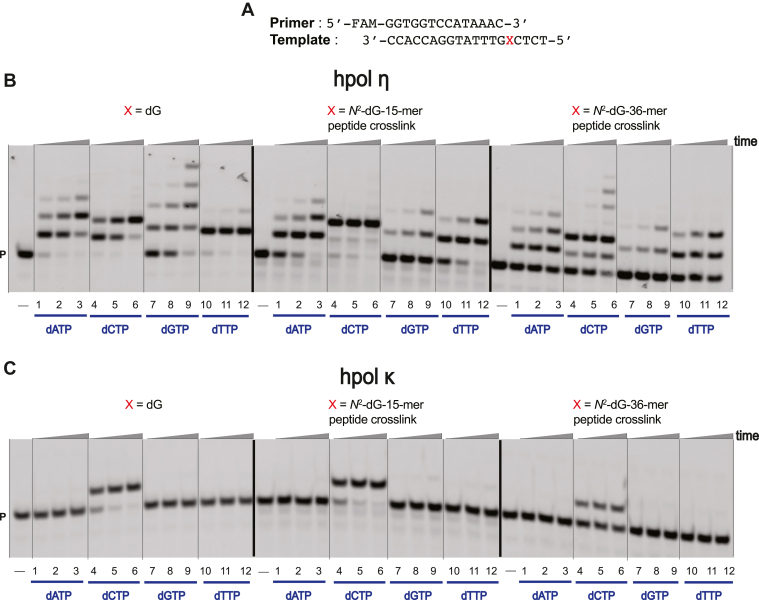
**Single nucleotide insertion by hpol η and hpol κ.***A*, 14-mer primer and 19-mer template sequences, where X is dG or *N*^2^-dG-15-mer peptide cross-link or *N*^2^-dG-36-mer peptide cross-link. Reactions were done in the presence of: *B*, 5 nM hpol η; *C*, 5 nM hpol κ. All reactions were done at 37 °C for 0-, 5-, 10-, or 30-min. Lanes 2–4*B*, *C* for dATP; lanes 5–7*B*, *C* for dCTP; lanes 8–10*B*, *C* for, dGTP; lanes 11–13*B*, *C* for dTTP with X is dG or *N*^2^-dG-15-mer peptide cross-link or *N*^2^-dG-36-mer peptide cross-link. P, FAM-labeled 14-mer DNA primer.

Single-nucleotide insertion assays were carried out using a 14-mer primer and respective unmodified and cross-linked templates in the presence of all four of the individual dNTPs (Fig. 5*A*). As observed earlier in our laboratory and by others (*e.g.*, Ghodke *et al.* (39)), hpol η is prone to misincorporation at high dNTP concentrations and to insert multiple copies of a single dNTP. hpol η preferentially added dCTP across from (unmodified) dG, with the incorporation preference dCTP > dATP > dTTP > dGTP (Fig. 5*B*, lanes 1–12). For both the *N*^2^-dG peptide cross-links, the incorporation preference was also dCTP > dATP > dTTP > dGTP (Fig. 5*B*, lanes 1–12, X is *N*^2^-dG-15-mer or 36-mer peptide cross-link).

With hpol κ, only dCTP incorporation was observed for all the templates (Fig. 5*C*). hpol κ had a slower rate of incorporation of dCTP across from *N*^2^-dG-36-mer peptide cross-link as compared with *N*^2^-dG-15-mer peptide cross-link (Fig. 5*C*, lanes 1–12, X is *N*^2^-dG-36-mer peptide cross-link). Overall, hpol κ showed faithful bypass of *N*^2^-dG-peptide cross-links irrespective of the bulk of the adduct.

### Miscoding potential of *N*^2^-dG oligonucleotide peptide cross-links

**Figure 6:**
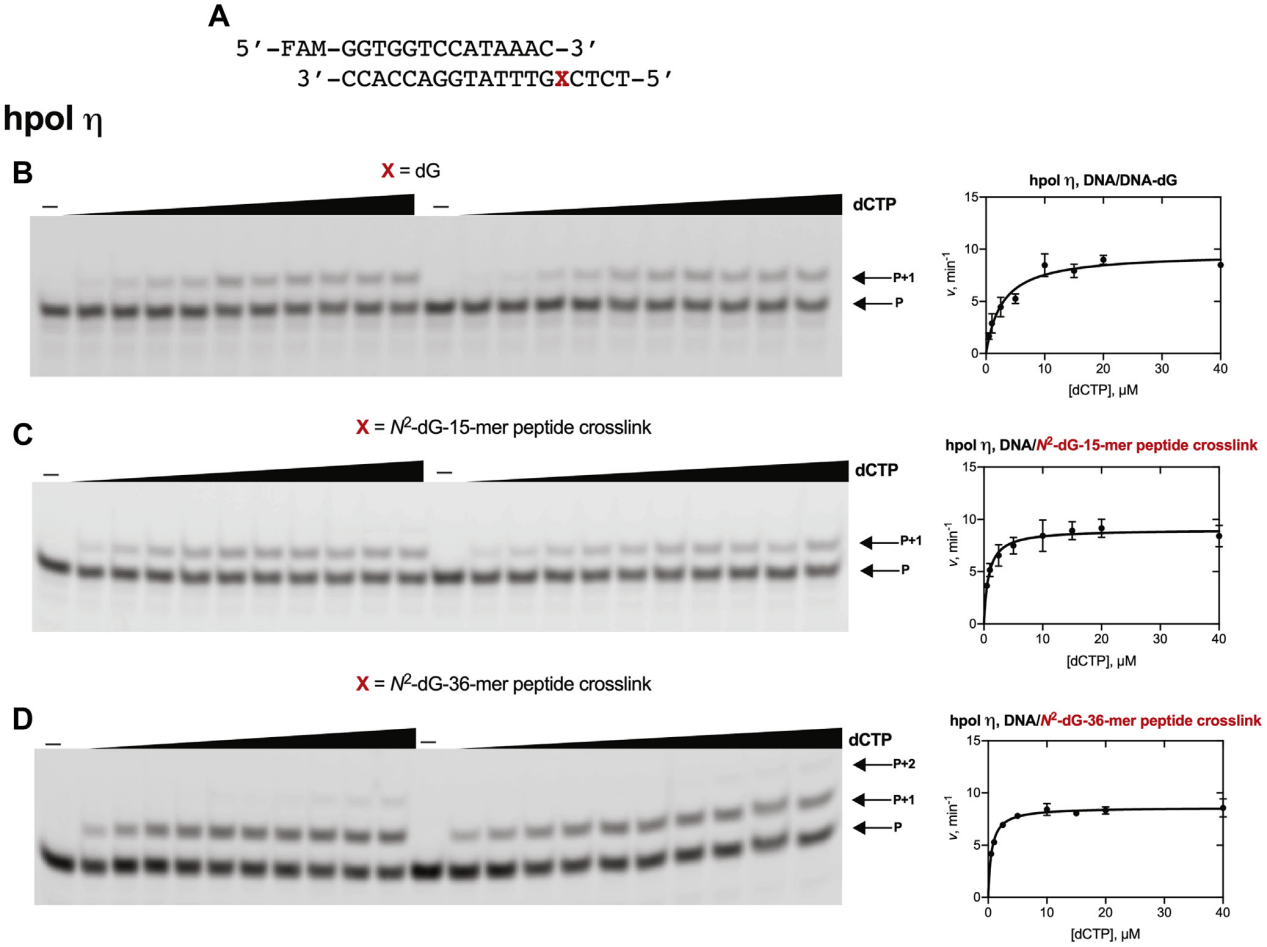
**Steady-state kinetic analysis of dCTP insertion by hpol η.***A*, 14-mer primer and 19-mer DNA template sequences, where X is dG or *N*^2^-dG-15-mer or 36-mer peptide cross-link. Reactions were done using hpol η: *B*, 0.75 nM; *C*, 0.75 nM and *D*, 1 nM. Varying concentrations of dCTP were used (0.5–40 μM). All reactions were carried out in duplicate at 37 °C for 5 min. Data points are shown as means ± SD. See Table 1 for *k*_cat_ and *K*_m_ values (fit to a hyperbolic equation in Prism) and Table S1 for the oligonucleotide sequences used. P, FAM-labeled 14-mer DNA primer.

**Figure 7:**
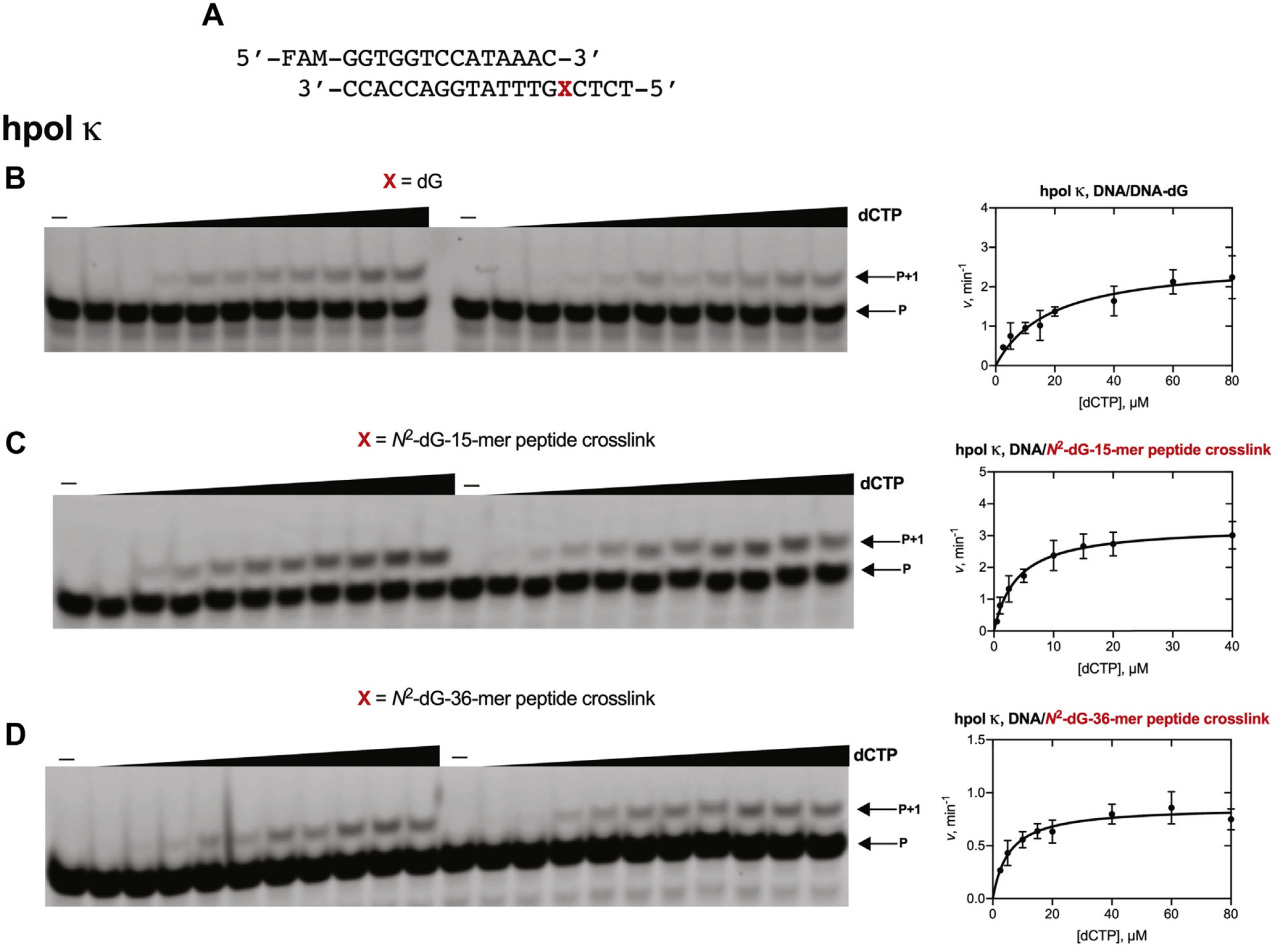
**Steady-state kinetic analysis of dCTP insertion by hpol κ.***A*, 14-mer primer and 19-mer DNA template sequences, where X is dG or *N*^2^-dG-15-mer or 36-mer peptide cross-link. Reactions were done using hpol κ: *B*, 2 nM; *C*, 2 nM and *D*, 2.5 nM. Varying concentrations of dCTP were used (*B*, 2.5–80 μM; *C*, 0.5–40 μM; *D*, 2.5–80 μM). All reactions were carried out in duplicate at 37 °C for 5 min. Data points are shown as means ± SD. See Table 1 for *k*_cat_ and *K*_m_ values (fit to a hyperbolic equation in Prism) and Table S1 for the oligonucleotide sequences used. P, FAM-labeled 14-mer DNA primer.

To determine the frequency of misincorporation across from *N*^2^-dG-peptide cross-links, steady-state kinetic analysis was done using individual dNTPs at varying concentrations with both hpol η and hpol κ (Figs. 6, 7, and S10–S12 and Table 1). The steady-state kinetics of insertion of the correct base (*i.e.*, dCTP) by hpol η and κ are shown in Figures 6 and 7. The steady-state kinetics of misincorporations (*i.e.*, dATP, dGTP, and dTTP) are shown in Figures S10–S12.

**Table 1:** Steady-state kinetic analysis of insertion opposite *N*^2^-dG-15-mer and 36-mer peptide cross-links

5′-FAM-GGTGGTCCATAAAC-3′ 3′-CCACCAGGTATTTGXCTCT-5′						
Polymerase	X	dNTP	*k*_cat_, min^−1^	*K*_m_, μM	*k*_cat_/*K*_m_, μM^−1^ min^−1^	*f*^a^
hpol η	dG	dCTP	9.6 ± 0.4	2.7 ± 0.5	3.4 ± 0.5	1
	*N*^2^-dG-15-mer peptide cross-link	dCTP	9.0 ± 0.3	0.79 ± 0.15	11.4 ± 1.9	1
	*N*^2^-dG-36-mer peptide cross-link	dCTP	8.6 ± 0.1	0.57 ± 0.05	15.0 ± 1.3	1
	dG	dATP	1.3 ± 0.1	3.0 ± 1.1	0.43 ± 0.13	0.13
	*N*^2^-dG-15-mer peptide cross-link	dATP	0.81 ± 0.04	2.3 ± 0.8	0.34 ± 0.10	0.030
	dG	dGTP	0.94 ± 0.11	92 ± 27	0.010 ± 0.001	0.003
	*N*^2^-dG-15-mer peptide cross-link	dGTP	0.24 ± 0.01	4.7 ± 1.1	0.05 ± 0.01	0.004
	dG	dTTP	4.5 ± 0.5	67 ± 16	0.067 ± 0.009	0.020
	*N*^2^-dG-15-mer peptide cross-link	dTTP	2.9 ± 0.3	78 ± 15	0.036 ± 0.04	0.003
hpol κ	dG	dCTP	2.7 ± 0.3	18.7 ± 5.6	0.14 ± 0.03	1
	*N*^2^-dG-15-mer peptide cross-link	dCTP	3.3 ± 0.2	3.9 ± 0.8	0.84 ± 0.14	1
	*N*^2^-dG-36-mer peptide cross-link	dCTP	0.86 ± 0.05	5.4 ± 1.3	0.15 ± 0.03	1

a Misincorporation frequency (*f*) = (*k*_cat_/*K*_m_)_incorrect_/(*k*_cat_/*K*_m_)_correct_.

The specificity constant (*k*_sp_ (42), i.e., *k*_cat_/*K*_m_) for the insertion of the correct nucleotide (dCTP) by hpol η opposite the *N*^2^-dG-15-mer peptide cross-link was 11.4 ± 1.9 μM^−1^ min^−1^ (Table 1), 3.4-fold higher than for insertion of dCTP opposite unmodified template (3.4 ± 0.5 μM^−1^ min^−1^) (Table 1). The specificity constant for the insertion of the correct nucleotide, dCTP, by hpol η opposite to the *N*^2^-dG-36-mer peptide cross-link was 15.0 ± 1.3 μM^−1^ min^−1^ (Table 1), 4.4-fold higher than for insertion of dCTP opposite the unmodified template. hpol η inserted all four dNTPs across from peptide cross-links. The specificity constants for misincorporation of dATP and dTTP for the *N*^2^-dG-15-mer peptide cross-link were 1.2- and 1.8-fold lower compared with the unmodified template. The specificity constant for dGTP misinsertion across from the *N*^2^-dG-15-mer peptide cross-link was 5-fold higher compared with the unmodified template.

The specificity constant for the insertion of correct nucleotide, dCTP, by hpol κ with the *N*^2^-dG-15-mer peptide cross-link was 0.84 ± 0.14 μM^−1^ min^−1^ (Table 1), 6-fold higher than for insertion of dCTP opposite unmodified template (0.14 ± 0.03 μM^−1^ min^−1^) (Table 1). Interestingly, the specificity constant for the insertion of correct nucleotide (dCTP) by hpol κ opposite the *N*^2^-dG-36-mer peptide cross-link was 0.15 ± 0.03 μM^−1^ min^−1^ (Table 1), essentially the same as for insertion of dCTP opposite dG in the unmodified template.

Comparison of the specificity constants indicated that the 15-mer peptide cross-link did not increase the misinsertion frequency compared with dG.

### LC-ESI-MS/MS analysis of hpol η primer extension past the *N*^2^-dG-15-mer peptide cross-link

As discussed earlier, hpol η inserted all four dNTPs opposite the *N*^2^-dG-15-mer peptide cross-link (Table 1). MS analysis was performed to define other miscoding events occurring with hpol η. Full-length extension reactions were carried out using a 2′-deoxyuridine (dU)-containing 14-mer primer and the unmodified and *N*^2^-dG-15-mer peptide-cross-linked templates. After full-length extension by hpol η, the reaction mixtures were treated with uracil-DNA glycosylase (UDG) and piperidine (43) to cleave the oligonucleotide to a length that could be analyzed by LC-MS/MS (Fig. S13). The fully extended products and relative yields are summarized in Table 2.

**Table 2:** Summary of products of extension of template–primer complexes by hpol η analyzed by LC-ESI-MS/MS

Primer: 5′-FAM-GGTGGTCCAUAAAC-3′ Template: 3′-CCACCAGGTATTTGXCTCT-5′				
X	Sequence	Yield (%)	Observed *m/z* (charge)	Base added
dG	5′-pAAAC**C**GAGA-3′	25%	939.36 (−3)	C
	5′-pAAAC**C**GAGAA-3′	71%	1043.64 (−3)	C, blunt end addition of A
	5′-pAAAC**C**GAGAAA-3′	4%	1148.00 (−3)	C, blunt end addition of AA
*N*^2^-dG-15-mer peptide cross-link	5′-pAAAC**C**GAGA-3′	18%	939.36 (−3)	C
	5′-pAAAC**C**GAGAA-3′	77%	1043.82 (−3)	C, blunt end addition of A
	5′-pAAAC**C**GAGAAA-3′	5%	1148.36 (−3)	C, blunt end addition of AA

hpol η yielded only error-free products for both the unmodified and cross-linked template, either with or without blunt end addition of A. For the unmodified template–primer complex, the major ions at *m/z* 939.36 (−3), 1043.64 (−3), and 1148.00 (−3) were observed (Figs. S14–S16; Tables S4–S6). For the *N*^2^-dG-15-mer peptide cross-link template–primer complex, *m/z* 939.36 (−3) is indicative of insertion of the correct base C (Fig. 8, Table S7). Ions at *m/z* 1043.82 (−3) and 1148.36 (−3) were also observed, indicating insertion of the correct base C with blunt end addition of A or two As (Figs. S17 and S18; Tables S8 and S9). Overall, the MS analysis revealed only an error-free bypass of the *N*^2^-dG-15-mer peptide cross-link.

**Figure 8:**
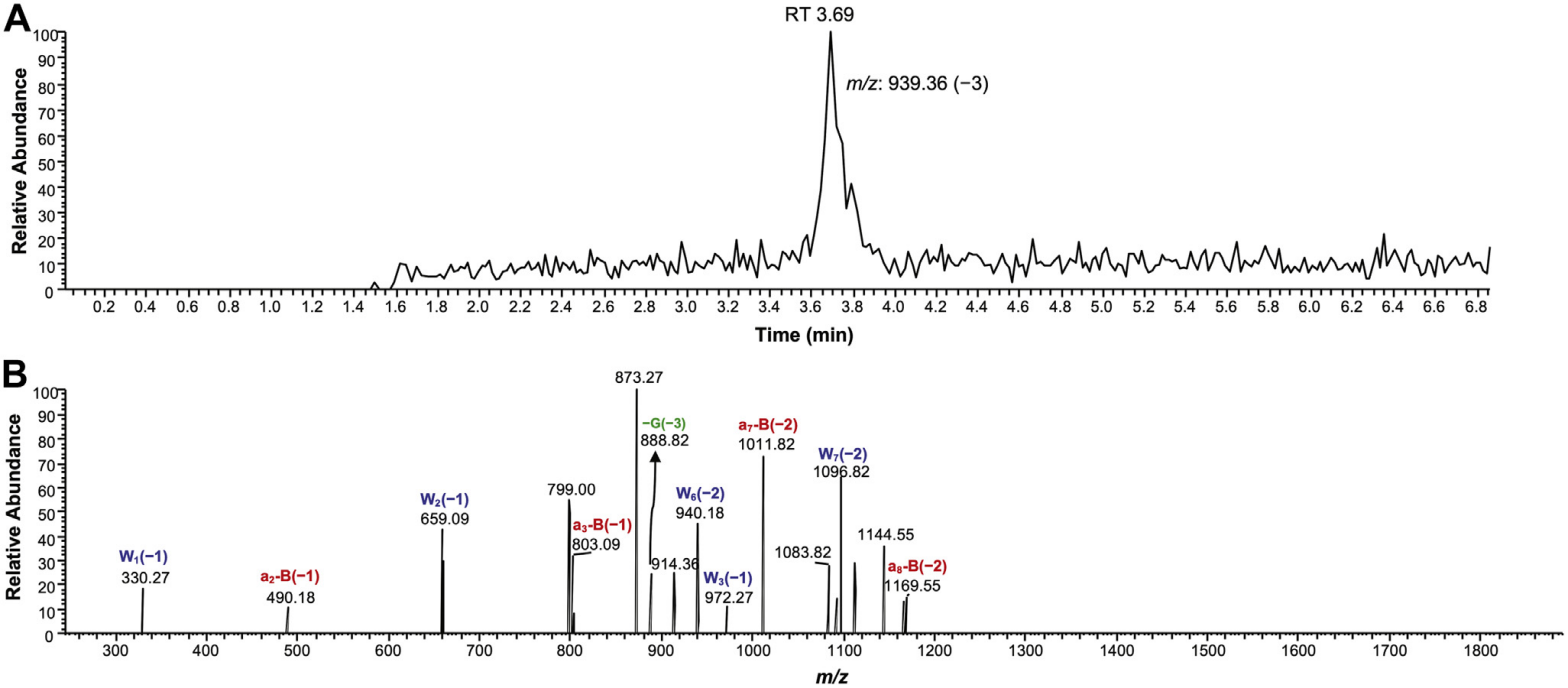
**LC-ESI-MS/MS sequence analysis of full-length extension reactions for *N***^**2**^**-dG-15-mer peptide cross-link by hpol η in the presence of dNTPs.***A*, Extracted ion chromatogram and *B*, CID spectrum of *m/z* 939.36 (−3, *t*_R_ 3.69 min) for *N*^2^-dG-15-mer peptide DNA cross-link. See Table S7 for fragment assignments.

## Discussion

Our current working hypothesis is that the AGT-DNA cross-links induced by 1,2-dibromoethane and other bis-electrophiles (35, 44) undergo proteolysis and generate DNA–peptide cross-links that can be bypassed by TLS polymerases (Fig. 1). Accordingly, we considered DNA–peptide cross-links formed at the *N*^2^-position of dG, generated from the active site of AGT (Cys-145) (35, 43). We focused on a 15-mer long AGT peptide cross-linked at the N2-position of dG in DNA and the major TLS polymerases hpol η and κ, which were considered to be the ones most likely involved in the bypass of DNA–peptide cross-links, based on our previous experience with bulky *N*^2^-dG adducts (45, 46) and with another cross-link (*N*^6^-dA) (39). Following initial results with the 15-mer, we extended some of the work to a 36-mer derived from the AGT active site, known to be cross-linked in biological settings (35). The chemical synthesis of the *N*^2^-dG DNA–peptide cross-links was achieved by coupling a thiol-containing oligonucleotide and dehydroalanine-modified 15-mer or 36-mer peptide (Fig. 2). As a result, oxidized *N*^2^-dG DNA-peptide cross-links were obtained (Fig. 2).

The enzymatic and MS studies were performed with this (oxidized form of the) *N*^2^-dG-oligonucleotide-peptide cross-link (Figs. 3, S8, and S9), which we encountered with both the 15- and 36-mer peptides. We have been unable to avoid what appears to be the artifactual oxidation of the thioether of Cys to the sulfone in this or work with the *N*^6^-dA cross-link (39) although we had not observed this species in earlier work on the isolation of the cross-linked peptide from DNA (34, 35, 37, 45). The only other differences we introduced, compared with the natural cross-link, was in the AGT peptide, is that we converted Cys-150 to prevent competition with Cys-145 and changed Lys-125 and Met-134 to leucine because of nucleophilic interference in the synthetic procedure we used.

Early studies from the Lloyd laboratory showed that bacterial DNA polymerases could efficiently bypass 4-mer- and 12-mer peptides linked to DNA by a ring-opened γ-hydroxypropano dG adduct (47, 48). Since then, several studies have been done with human TLS polymerases that can bypass DNA–peptide cross-links (19, 49, 50), although the number of reports is relatively small. However, these have involved shorter peptides (10-mers), and in one study a 23-mer posed a strong block to replication by hpol η, ι, and κ (50). Almost all of these studies used artificial linkages or peptides from model proteins, with no clear connection to actual DNA–protein cross-link structures or biological phenomena.

Among the various human TLS polymerases, hpol η and κ have been studied with various *N*^2^-dG DNA adducts (45, 46, 51, 52). Our full-length primer extension assays showed that hpol η and κ tolerated both the *N*^2^-dG-15-mer and 36-mer peptide cross-links (Fig. 4). These results indicate that both polymerases can tolerate the 36-amino acid residue bulk found in the minor groove of DNA. Single-nucleotide incorporation assays indicated that only hpol η misincorporated individual dNTPs across from unmodified template, the *N*^2^-dG-15-mer, and the 36-mer peptide cross-links (Fig. 5). hpol κ showed the only incorporation of dCTP across from unmodified template and the *N*^2^-dG-15-mer and 36-mer peptide cross-links, indicating high-fidelity bypass of the oxidized oligonucleotide–peptide cross-links (Fig. 5).

We analyzed hpol η-mediated misincorporation levels by comparing specificity constants for incorporation of individual dNTP across from unmodified, *N*^2^-dG-15-mer, and 36-mer peptide cross-link templates. With the *N*^2^-dG-15-mer peptide cross-link, hpol η inserted dATP 33-fold, dGTP 228-fold, and dTTP 316-fold less efficiently than dCTP (Figs. 6 and S10–12 and Table 1) with an overall misincorporation frequency less than that observed for dG. The steady-state kinetic results indicate a very low level of misincorporation at the site of the *N*^2^-dG-15-mer peptide cross-link by hpol η.

We also compared hpol η-mediated correct base incorporation opposite *N*^2^-dG-15-mer as well as 36-mer peptide cross-link templates. The steady-state kinetic results suggest that the specificity constant for incorporation of dCTP across from *N*^2^-dG-15-mer peptide cross-link template was increased by 3.5-fold as compared with dG (Table 1). Surprisingly, the *N*^2^-dG-36-mer peptide cross-link template showed even faster insertion. As a result, the 36-amino acid bulk had only a minor effect on extension of the primer (Fig. 4) but did not attenuate single dCTP incorporation rates.

In the case of hpol κ (Fig. 7), the steady-state kinetic specificity constant for incorporation of dCTP across from *N*^2^-dG-15-mer peptide cross-link template was increased 6-fold compared with dG, and the specificity constant with the *N*^2^-dG-36-mer peptide cross-link template was nearly identical to the rate measured with dG (Table 1). These findings are consistent with previous studies with hpol κ and bulky *N*^2^-dG adducts (46, 51), in which alkyl and polycyclic aromatic hydrocarbon adducts had only small effects on the efficiency of polymerization or on misincorporation (46).

MS analysis of bypass of the *N*^2^-dG-15-mer peptide cross-link by hpol η showed products with the only insertion of the correct base C across from the adducted site (with or without blunt end addition of A, Table 2, Figs. 8, S17, and S18). Previous studies of our own and others have also shown the efficient bypass of some smaller *N*^2^-dG DNA adducts from the minor groove of DNA (46, 52). As discussed earlier, in steady-state kinetics the extent of misincorporation observed opposite the *N*^2^-dG-15-mer peptide cross-link was less than for dG (Table 1). In contrast, our own studies with *N*^6^-dA-oligonucleotide–peptide cross-links showed 37% of overall misincorporation by hpol η (39).

On the basis of both steady-state kinetics and mass spectral analysis, we conclude that other bases (*i.e.*, A, G, and T) can be inserted across from the *N*^2^-dG-15-mer peptide cross-link (Table 1) but that the misincorporation frequency for hpol η is not higher than opposite unmodified dG. Further, if a misincorporation occurs, the enzyme is not able to extend the primer further, so mutations should not result.

In conclusion, the steady-state kinetics and mass spectral analysis of extended primers revealed only error-free products while bypassing the *N*^2^-dG-15-mer AGT peptide cross-link. In contrast, our own studies with *N*^6^-dA-oligonucleotide–peptide cross-links showed 37% of overall misincorporation by hpol η (39). The lack of miscoding with such bulky *N*^2^-dG peptides was somewhat surprising, but the ability of these enzymes to bypass such large peptides was also very unexpected. The formula weight of the 36-mer is >3500, and an object of future investigation is to understand how the structures of these polymerases can accommodate the bulk so well from the minor groove of DNA.

## Experimental procedures

### Reagents

2-F-dI phosphoramidite was purchased from Glen Research. Unmodified oligonucleotides and 6-carboxyfluorescein (FAM)-labeled oligonucleotide primers were purchased from Integrated DNA Technologies (IDT). The 15- and 36-mer peptides were purchased from New England Peptides. C_18_ Sep-Pak columns were purchased from Waters. Other chemical reagents were from Sigma-Aldrich. hpol η (catalytic core of 1–432 amino acids) and hpol κ (19–526 amino acids) were expressed in *E. coli* and purified as previously reported (53, 54). Unlabeled dNTPs and UDG were purchased from New England Biolabs. Micro Biospin-6 columns were purchased from Bio-Rad.

### Synthesis of 15-mer and 36-mer dehydroalanine-modified peptides (dha peptides)

The synthesis, purification, and characterization of the 15-mer dehydroalanine-modified peptide were done as published previously (39).

The following procedure was used for the 36-mer dehydroalanine-modified peptide (Fig. S1). K_2_CO_3_ (4 mg) was dissolved in 100 μl of nuclease-free H_2_O and added to 10 mg of 36-mer peptide (Ac-PLAARAVGGALRGNPVPILIPCHRVVSSSGAVGNYS-NH_2_). MSH (1.5 mg) was dissolved in 100 μl of anhydrous *N*,*N*-dimethylformamide and added dropwise into the 36-mer peptide solution, which was then incubated on ice for 20 min with vortex mixing after every 3 min. After 20 min, the reaction mixture was diluted in 1 ml of mobile phase A (95% H_2_O, 5% CH_3_CN, 0.1% HCO_2_H; v/v/v) and purified by HPLC using a Phenomenex octadecylsilane (C_18_) semipreparative HPLC column (10 mm × 250 mm, 5 μm). The sample was eluted at a flow rate of 3 ml min^−1^, with UV detection at 240 nm (to avoid saturation of the signal). Buffers consisted of mobile phases A (see above) and B (95% CH_3_CN, 5% H_2_O, 0.1% HCO_2_H; v/v/v). The following gradient was used: 0–5 min, 10% B; 5–20 min, 10–50% B; 20–25 min, 50–100% B; 25–30 min, 100% B and 30–32 min, 100–10% B (all v/v). The 36-mer dehydroalanine-modified peptide was eluted at approximately 18 min (Fig. S2), and the appropriate fractions were collected and lyophilized. The identity was confirmed (Fig. S3) by positive ion ESI nano-LC-MS analysis.

### Oligonucleotide synthesis

Solid-phase synthesis of the 2-F-dI containing oligonucleotide was done on a PerSeptive Biosystems Model 8909 DNA synthesizer. The oligonucleotides were synthesized on a 1-μmol scale using the appropriate CPG as solid support.

### Synthesis of *N*^2^-cystamine-dG oligonucleotide

The synthesis of the *N*^2^-cystamine-dG modified oligonucleotide was achieved on CPG (Fig. 2). The CPG-bound 2-F-dI containing oligonucleotide (5′-TCTCXGTTTATGGACCACC-3′, where X is 2-F-dI) was treated with cystamine-2 HCl (10 μl of 500 mM stock in DMSO) in a mixture of DMSO (86 μl) and diisopropylethylamine (4.4 μl), with incubation at 55 °C for 22 h to obtain the *O*^6^-(*p*-nitrophenylethyl) (NPE)-protected *N*^2^-cystamine-dG-modified oligonucleotide. Next, the supernatant was discarded carefully, and the CPG was washed with DMSO (2 ml), followed by CH_3_CN (3 ml), and then air-dried. NPE deprotection of *N*^2^-cystamine-dG modified oligonucleotide was carried out using 1 M solution of DBU in CH_3_CN (1 ml) for 2 h at room temperature with continuous shaking. After DBU treatment, the DBU solution was carefully removed and the CPG was washed with anhydrous CH_3_OH (2 ml) and with anhydrous CH_3_CN (3 ml) and then air-dried. Finally, the NPE-deprotected *N*^2^-cystamine-dG-modified oligonucleotide CPG was treated with 1 ml of 0.4 M NaOH (in CH_3_OH) at room temperature overnight with continuous stirring. After completion of the final deprotection step, the CPG was sonicated for 5 min. Further, the supernatant was collected in an Eppendorf tube and the CPG was washed with H_2_O (400 μl); the supernatant was collected in the same tube. The collected supernatant was neutralized to pH 7.0 with 10% glacial CH_3_CO_2_H (v/v) and concentrated in vacuo using a centrifugal evaporator. Next, a dried DNA pellet was resuspended in 300 μl of nuclease-free water.

HPLC purification of *N*^2^-cystamine-dG modified oligonucleotide was carried out using mobile phase A (0.1 M triethylammonium acetate (TEAA), pH 7.0) and mobile phase B (0.1 M triethylammonium acetate (TEAA), and CH_3_CN, 1:1, v/v) using a Phenomenex Clarity Oligo-RP (C_18_) column (150 mm × 10 mm, 5 μm) at room temperature. UV detection was at 260 nm. The following gradient program (v/v) was used with a flow rate of 3 ml min^−1^: started at 17% B, continued for 5 min, then increased to 40% B over 20 min, then increased to 100% B at 21 min, held at 100% B for 5 min, and re-equilibrated for 4 min at 17% B (all v/v). The oligonucleotide eluted at 16.5 min (Fig. S4). The desired oligonucleotide fractions were collected and concentrated with a centrifugal evaporator. Finally, the purified oligonucleotide pellet was resuspended in 20 ml of 10 mM Tris-HCl buffer (pH 8.0) containing 1 mM EDTA and 300 mM NaCl (TEN buffer) and desalted using a C_18_ Sep-Pak column. The integrity of the *N*^2^-cystamine-dG modified oligonucleotide was confirmed by MALDI-TOF MS (Fig. S5).

### Synthesis of *N*^2^-dG-15-mer and 36-mer peptide cross-links

Reduction of the disulfide bond of *N*^2^-cystamine-dG oligonucleotide yielded the *N*^2^-(2-thioethyl)-dG-modified oligonucleotide (Fig. 2). DTT (2.5 μl of a 500 mM solution), HEPES buffer (1 M, pH 7.3, 10 μl), and water (14 μl) were added to the solution of the oligonucleotide (24 μl, 6 nmol) (total 50 μl). The reaction mixture was incubated at 37 °C for 2 h. The reaction mixture was further precipitated: 3 M NaCl (30 μl) and H_2_O (220 μl) were added and the mixture was mixed with a vortex device. Further, C_2_H_5_OH (900 μl) was added to the same tube, vortex mixing was done, and the sample was stored at –80 °C for overnight. Next, the mixture was centrifuged (21,000*g*, 30 min at 4 °C), and the supernatant was carefully removed. The pellet was washed with absolute C_2_H_5_OH (100 μl). After centrifugation at 21,000*g* at 4 °C for 20 min, the supernatant was removed. Finally, the oligonucleotide pellet was air-dried.

The dehydroalanine peptide (7 mg of the 15-mer or 5 mg of the 36-mer) was added to 40 μl of 100 mM Na_2_CO_3_ (pH 9.2), mixed with a vortex device, and centrifuged. This peptide solution was added to the dry oligonucleotide pellet. An additional 10 μl of 100 mM Na_2_CO_3_ (pH 9.2) and 10 μl of 10% CH_3_CN (v/v) were used to dissolve the rest of the peptide. This solution was added to the oligonucleotide solution again and mixed with a vortex device. The reaction mixture (total 60 μl) was incubated at 37 °C for overnight. After ∼12 h, reaction mixtures were loaded directly for further purification by gel electrophoresis. Products were separated using a 20% polyacrylamide gel electrophoresis (PAGE) (7 M urea), at 55 W for 3 h (Fig. S6).

The desired oligonucleotide–peptide cross-link bands were located by brief exposure to a UV lamp (260 nm). The desired oligonucleotide–peptide cross-links (which migrated more slowly than the control oligonucleotide) were extracted from gel pieces by soaking in TEN buffer at 4 °C overnight. The vial containing the gel fragment was shaken for 1 h to release the oligonucleotide–peptide cross-link into the buffer. The buffer solution from the vial was collected in a clean Falcon tube. This extraction step was repeated, and the buffer fractions were combined and desalted using a C_18_ Sep-Pak column.

The integrity of the oligonucleotide–peptide cross-links was confirmed by MALDI MS (Fig. 3), as well as positive ion ESI nano-LC-MS/MS (Figs. S8 and S9). An additional mass of 32 a. m. u. was found, indicative of apparent oxidation to the sulfone.

For nano-LC-MS/MS, the desired DNA–peptide cross-links were hydrolyzed using HF to obtain a peptide adducted with only the base guanine (Fig. S7). The desired oligonucleotide–peptide cross-links (125–150 pmol each) were dried and suspended in HF (48%, 50 μl) and incubated at 4 °C for 16 h. Next, the hydrolyzed samples were dried under a stream of nitrogen and resuspended in anhydrous CH_3_OH (50 μl). These samples were dried again under a stream of nitrogen and the same step repeated once. The dried pellet of hydrolyzed oligonucleotide–peptide cross-links was dissolved in 0.1% HCO_2_H (20 μl, v/v), shaken for 10 min, and centrifuged for 5 min at 23 °C (21,000*g*). Finally, nucleobase-adducted peptides (without any purification) were analyzed using a nanoLC Ultra system (Eksigent Technologies) interfaced with an LTQ Orbitrap XL mass spectrometer (Thermo Scientific) in the positive ion mode as described previously (41), except for the separation conditions: a linear gradient increased from 2% to 45% solvent B over a period of 0–45 min, increased from 45% to 95% solvent B over a period of 45–50 min, held at 95% solvent B over a period of 50–60 min, decrease from 95% to 2% solvent B over a period of 60–62 min, and column was equilibrated at 2% solvent B over a period of 62–72 min (all v/v).

MS analysis showed that the fragmentation patterns of oligonucleotide–peptide cross-links are consistent with the additional mass of 32 a. m. u. indicating the oxidized form of sulfur (sulfone) (Figs. S8 and S9; Tables S2 and S3).

### Full-length extension assays

A primer–template complex containing a FAM-labeled oligonucleotide primer (14-mer) and an unmodified or *N*^2^-dG-15-mer- or 36-mer peptide-cross-linked templates were annealed (1:1 M ratio) at 95 °C for 5 min, followed by slow cooling overnight (Fig. 4). The full-length extension reactions were carried out in 50 mM Tris-HCl buffer (pH 7.5) containing 50 mM NaCl, 5 mM MgCl_2_, 5% glycerol (v/v), 5 mM DTT, and 50 μg ml^−1^ bovine serum albumin (BSA) at 37 °C. The final concentration of primer–template complex was 120 nM. Concentrations of 20 nM hpol η and hpol κ were used to obtain extended primers. Reactions were initiated by adding a 1 μl equimolar mixture of dNTPs (final concentration 250 μM) to a total volume of 25 μl. Aliquots (3.5 μl) of reaction mixtures were taken at different time points (0, 5, 10, 30, 60, and 120 min) and quenched with 6.5 μl of 10 mM EDTA (pH 8.0) in 95% deionized formamide (v/v). Products (5 μl) were separated using 20% PAGE (7 M urea), and results were visualized using a Typhoon scanner (GE Healthcare) and analyzed by ImageJ software.

### Single-nucleotide insertion assays

A primer–template oligonucleotide complex containing a FAM-labeled oligonucleotide primer (14-mer) and an unmodified or *N*^2^-dG-15-mer as well as 36-mer peptide cross-linked templates were annealed (1:1 M ratio) at 95 °C for 5 min, followed by slow cooling overnight (Fig. 5). All single-nucleotide insertion reactions were performed using 50 mM Tris-HCl buffer (pH 7.5) containing 50 mM NaCl, 5 mM MgCl_2_, 5% glycerol (v/v), 5 mM DTT, and 50 μg ml^–1^ BSA at 37 °C. The final concentration of primer–template complex was 120 nM. Concentrations of 5 nM hpol η and hpol κ were used to obtain extended primers. The reactions were initiated by addition of 1 μl of each individual dNTP (final concentration 100 μM) to a total volume of 25 μl. Aliquots (3.5 μl) of reaction mixtures were taken at each time point (0, 5, 10, and 30 min) and quenched with 6.5 μl of 10 mM EDTA (pH 8.0) in 95% deionized formamide (v/v). Products were separated (5 μl) using 20% PAGE (7 M urea), and results were visualized using a Typhoon scanner (GE Healthcare) and analyzed by ImageJ software.

### Steady-state kinetics

A primer–template complex containing a FAM-labeled oligonucleotide primer (14-mer) and an unmodified or *N*^2^-dG-15-mer or 36-mer peptide cross-linked template were annealed (1:1 M ratio) at 95 °C for 5 min, followed by slow cooling overnight (Table 1, Figs. 6, 7 and S10–S12). All steady-state kinetic reactions were performed using 50 mM Tris-HCl buffer (pH 7.5) containing 50 mM NaCl, 5 mM MgCl_2_, 5% glycerol (v/v), 5 mM DTT, and 50 μg ml^-1^ BSA at 37 °C. The final concentration of primer–template complex was 120 nM. Desired concentrations of hpol η (ranging from 0.75 to 3 nM) and hpol κ (ranging from 2 to 2.5 nM) were used to maintain steady-state kinetics of each dNTP insertion (<30% product). Reactions were started by adding 1 μl of an individual dNTP stock solution, at each of ten different concentrations (in some cases only eight concentrations were used in plotting because product formation was >30%), to a total volume of 5 μl. Aliquots (3.5 μl) of reaction mixtures were taken at respective time points and quenched with 6.5 μl of 10 mM EDTA (pH 8.0) in 95% deionized formamide (v/v). Products were separated using 20% PAGE (7 M urea), and results were visualized using a Typhoon scanner (GE Healthcare) and analyzed by ImageJ software. Data points are shown as means ± SD (range) from two independent experiments; see Table 1 for *k*_cat_ and *K*_m_ values and estimated using fit to a hyperbolic equation in Prism software (GraphPad, San Diego, CA). Hyperbolic fitting was done to obtain *k*_cat_/*K*_m_ (*k*_sp_) and *k*_cat_ directly and then dividing to obtain *K*_m_ (42).

### LC-ESI-MS/MS: full-length extension assays followed by UDG and piperidine treatment

A 2′-dU-containing FAM-labeled 14-mer primer (5′-GGTGGTCCA**U**AAAC-3′) and an unmodified or *N*^2^-dG-15-mer cross-linked templates were annealed (1:1 M ratio) at 95 °C for 5 min followed by slow cooling overnight (Fig. S13) (43, 55). The full-length extension reactions were carried out under same reaction conditions as described in full-length extension reactions, except that the final concentrations were 2.5 μM primer–template complex and 0.75 μM hpol η, in a total reaction volume of 85 μl. Reactions were carried out in the presence of a mixture of dNTPs (final concentration 1 mM; *i.e.*, 250 μM of each dNTP) at 37 °C for 4 h. Reactions were terminated using Micro Biospin-6 column separations to extract Mg^2+^ and dNTPs. The resulting products were treated with 25 units of UDG at 37 °C for 4 h, followed by 0.25 M piperidine at 95 °C for 1 h. The reaction mixture was dried by lyophilization. The dried pellet was resuspended in 500 μl of nuclease-free H_2_O and taken to dryness by lyophilization. The dried pellet was resuspended in 25 μl of nuclease-free water for LC-ESI-MS/MS analysis.

### LC-ESI-MS/MS analyses

LC-ESI-MS/MS analyses were performed on a Finnigan LTQ mass spectrometer (Thermo Scientific) connected to an Acquity ultraperformance liquid chromatography (UPLC) system (Waters Corporation) as described previously (56), except for the UPLC conditions: Buffer A contained 10 mM NH_4_CH_3_CO_2_, 2% CH_3_CN, 1% CH_3_OH, and 97% H_2_O (v/v), pH 7.0, and buffer B contained 10 mM NH_4_CH_3_CO_2_, 95% CH_3_CN, 1% CH_3_OH, and 4% H_2_O (v/v), pH 7.0 (Table 2, Figs. 8 and S14–S18; Tables S4–S9). ESI settings: spray voltage 4.5 kV, sheath gas flow 40, auxiliary gas flow rate 15, sweep gas flow rate 5, capillary voltage −49 V, tube lens voltage −140 V, and capillary temperature 270 °C.

The fully extended products were identified (Table 2, Figs. 8 and S14–S18; Tables S4–S9) by comparing the observed CID fragments with the theoretical values using a Mongo Oligo Mass Calculator v2.06 (http://rna.rega.kuleuven.be/masspec/mongo.htm). The relative yields of extended products were calculated based on the peak areas of extracted ion chromatograms.

## Data availability

All data are contained within the article and the supporting information.

## Supporting information

This article contains supporting information (39).

## Conflict of interest

The authors declare that they have no conflict of interest with the contents of this article.

## Supporting information

List of oligonucleotide and peptide sequences used for the studies; synthetic strategy, HPLC purification, mass spectrum of 36-mer dehydroalanine peptide; HPLC purification, and MALDI mass spectrum of *N*^2^-cystamine-dG modified oligonucleotide; gel purification, flow chart for HF treatment and nanoLC-MS/MS, mass spectrum and fragmentation pattern of *N*^2^-dG-15-mer as well as 36-mer peptide cross-linked templates; PAGE analyses of steady-state kinetic analysis and the respective Michaelis–Menten plots; PAGE analyses of full-length extension assay for LC-ESI-MS/MS analysis; LC-ESI-MS/MS sequencing analysis of full-length extension reactions; respective extracted ion chromatograms and CID fragmentation spectrums.
