# Supplementary material for: DNA polymerases η and κ bypass N2-guanine-O6-alkylguanine DNA alkyltransferase cross-linked DNA-peptides
Source: J Biol Chem. 2021 Aug 28;297(4):101124. doi: 10.1016/j.jbc.2021.101124 (PMC8463853; doi:10.1016/j.jbc.2021.101124)
Supplement: Figures S1–S18 and Tables S1–S9 [file mmc1.docx]

**Supporting Information**

**Long peptides derived from DNA *N*^2^-guanine crosslinks with the DNA repair protein *O*^6^-alkylguanine DNA-alkyl transferase are bypassed by human DNA polymerases η and κ**

Pratibha P. Ghodke and F. Peter Guengerich

Department of Biochemistry, Vanderbilt University School of Medicine, Nashville, Tennessee 37232-0146 USA

**Table of Contents**

| Table S1 | List of oligonucleotide sequences and peptides | Page S-3 |
| --- | --- | --- |
| Figure S1 | Synthetic strategy for 15-mer and 36-mer dehydroalanine-modified peptides | Page S-4 |
| Figure S2 | HPLC purification of 36-mer dehydroalanine-modified peptide | Page S-4 |
| Figure S3 | ESI-MS analysis of 36-mer dehydroalanine-modified peptide | Page S-5 |
| Figure S4 | HPLC purification of *N*^2^-cystamine-dG-modified oligonucleotide | Page S-5 |
| Figure S5 | MALDI spectrum of *N*^2^-cystamine-dG-modified oligonucleotide | Page S-6 |
| Figure S6 | Gel purification of *N*^2^-dG DNA-peptide crosslinks | Page S-6 |
| Figure S7 | HF treatment and nano-LC-ESI-MS/MS of *N*^2^-dG DNA-peptide crosslinks | Page S-7 |
| Figure S8 | Extracted ion chromatogram and CID spectrum of *m/z* 927.56 (+2) of HF treated *N*^2^-dG-15-mer peptide crosslink | Page S-7 |
| Table S2 | Observed and theoretical CID fragments of *m/z* 927.56 (+2) of HF-treated *N*^2^-dG-15-mer peptide crosslink | Page S-8 |
| Figure S9 | Extracted ion chromatogram and CID spectrum of *m/z* 952.29 (+4) of HF- treated *N*^2^-dG-36-mer peptide crosslink | Page S-9 |
| Table S3 | Observed and theoretical CID fragments of *m/z* 952.29 (+4) of HF-treated *N*^2^-dG-36-mer peptide crosslink | Pages S10, S11 |
| Figure S10 | hpol η-mediated steady-state kinetic analysis with dATP | Page S-12 |
| Figure S11 | hpol η-mediated steady-state kinetic analysis with dGTP | Page S-12 |
| Figure S12 | hpol η-mediated steady-state kinetic analysis with dTTP | Page S-13 |
| Figure S13 | Full-length extension reactions using hpol η for LC-MS/MS sequence analysis | Page S-13 |
| Figure S14 | Extracted ion chromatogram for *m/z* 939.36 (−3, RT 3.66) and CID spectrum of *m/z* 939.36 (−3) for control template | Page S-14 |
| Table S4 | Observed and theoretical CID fragments of *m/z* 939.36 (−3) for control template | Page S-14 |
| Figure S15 | Extracted ion chromatogram for *m/z* 1043.64 (−3, RT 3.68) and CID spectrum of *m/z* 1043.64 (−3) for control template | Page S-15 |
| Table S5 | Observed and theoretical CID fragments of *m/z* 1043.64 (−3) for control template | Page S-16 |
| Figure S16 | Extracted ion chromatogram for *m/z* 1148.00 (−3, RT 3.68); (B) and CID spectrum of *m/z* 1148.00 (−3) for control template | Page S-17 |
| Table S6 | Observed and theoretical CID fragments of *m/z* 1148.00 (−3) for control template | Page S-18 |
| Table S7 | Observed and theoretical CID fragments of *m/z* 939.36 (−3) for *N*^2^-dG-15-mer peptide crosslink template | Page S-18 |
| Figure S17 | Extracted ion chromatogram for *m/z* 1043.82 (−3, RT 3.72) and CID spectrum of *m/z* 1043.82 (−3) for *N*^2^-dG-15-mer peptide crosslink template | Page S-19 |
| Table S8 | Observed and theoretical CID fragments of *m/z* 1043.82 (−3) for *N*^2^-dG-15-mer peptide crosslink template | Page S-20 |
| Figure S18 | Extracted ion chromatogram for *m/z* 1148.36 (−3, RT 3.72); (B) and CID spectrum of *m/z* 1148.36 (−3) for *N*^2^-dG-15-mer peptide crosslink template | Page S-21 |
| Table S9 | Observed and theoretical CID fragments of *m/z* 1148.36 (−3) for *N*^2^-dG-15-mer peptide crosslink template | Page S-21 |
| References |  | Page S-22 |

**Table S1**

List of oligonucleotide sequences and peptides

| **Code** | **Oligonucleotide sequences (5´-3´) and peptides** | ***m/z***  **(Calcd)** | ***m/z***  **(Found)** |
| --- | --- | --- | --- |
| Unmodified 19-mer | 5´-TCTCGGTTTATGGACCACC-3´ | From IDT*^a^* |  |
| Primer-I  (14-mer) | 5´-FAM-GGTGGTCCATAAAC-3´ | From IDT*^a^* |  |
| Primer-II  (LC-ESI-MS/MS) | 5´-FAM-GGTGGTCCA**U**AAAC-3´ | From IDT*^a^* |  |
| *N*^2^-cystamine-dG modified oligonucleotide | 5´-TCTCXGTTTATGGACCACC-3´  (X: *N*^2^-cystamine-dG) | 5890.0147 | 5891.6021 |
| 36-mer dha *^b^* peptide | Ac-P**L**AARAVGGA**L**RGNPVPILIP**dha**HRVV**S**SSGAVGNYS-NH_2_*^c^* | 1188.2993, (+3)  891.4763 (+4) | 1188.9969, (+3)  891.9981 (+4) |
| 15-mer dha peptide | Ac-PVPILIP**dha**HRVV**S**SS-NH_2_*^d^* | 1610.93 | 1611.0 *^d^* |
| *N*^2^-dG-15-mer peptide DNA crosslink | 5´-TCTCXGTTTATGGACCACC-3´  (X: oxidized 15-mer peptide) | 7459.8097 | 7458.8158  (MALDI) |
| *N*^2^-dG-15-mer peptide DNA crosslink | 5´-TCTCXGTTTATGGACCACC-3´  (X: oxidized 15-mer peptide) | 927.56, (+2) | 927.56, (+2)  (ESI-MS) |
| *N*^2^-dG-36-mer peptide DNA crosslink | 5´-TCTCXGTTTATGGACCACC-3´  (X: oxidized 36-mer peptide) | 952.29, (+4) | 952.29, (+4)  (ESI-MS) |

*^a^*Used from manufacturer (IDT) without further analysis.

*^b^*The abbreviation used is: dha, dehydroalanine.

*^c^*Lys-125 and Met-134 (in AGT) were changed to Leu and Cys-150 was changed to Ser because of reactivity in the synthesis of the crosslink.

*^d^*Ref. (1).


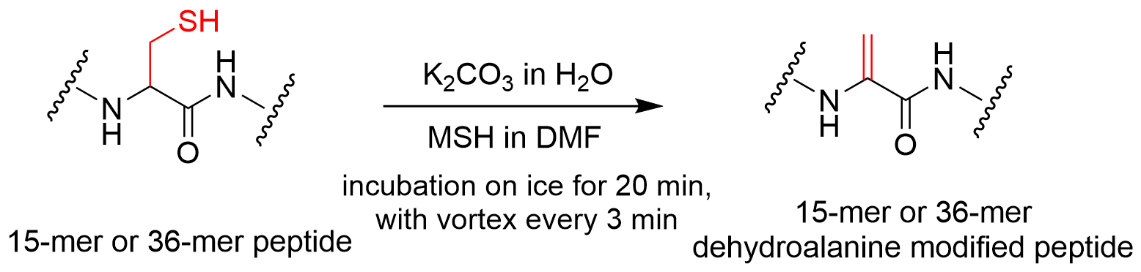


**Figure S1. Synthetic strategy for 15-mer and 36-mer dehydroalanine-modified peptides.**


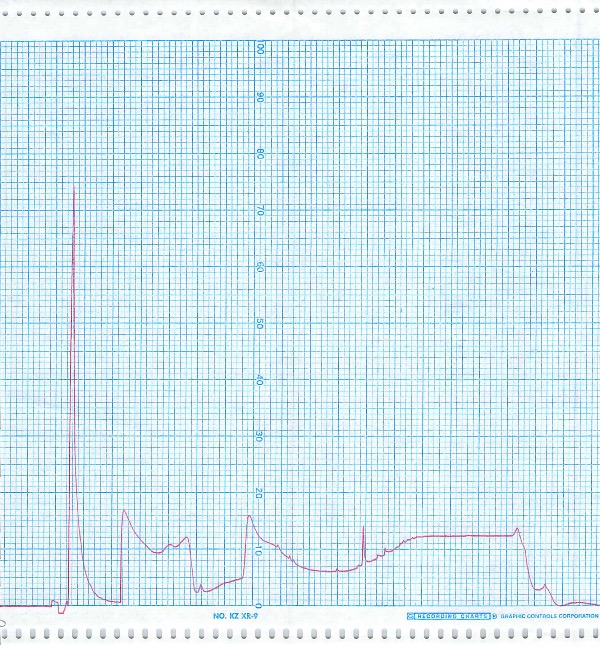


**Figure S2.** **HPLC purification of 36-mer dehydroalanine-modified peptide. The dehydroalanine peptide eluted at approximately 18 min (arrow).**


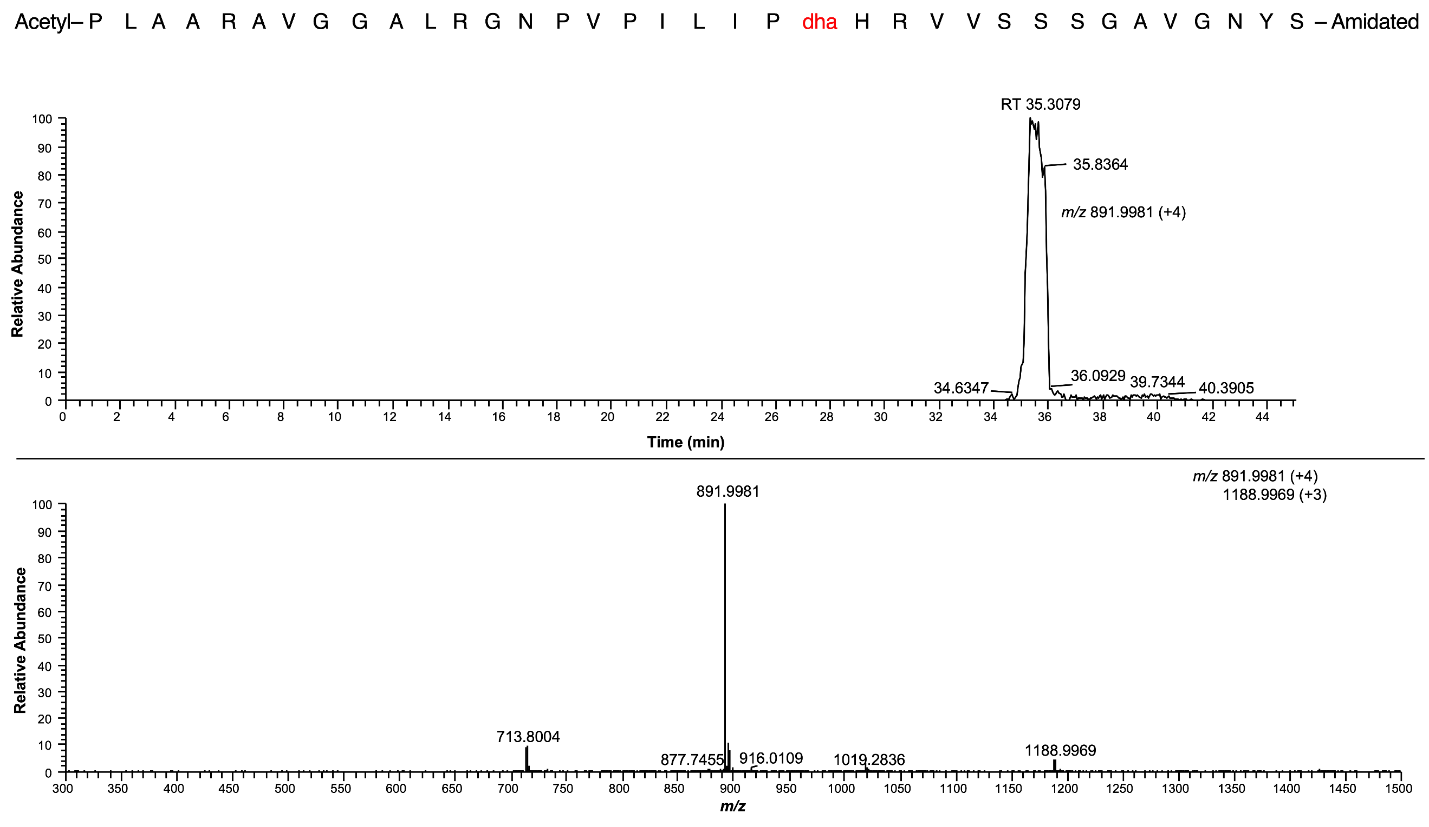


**Figure S3.** **ESI-MS analysis of 36-mer dehydroalanine-modified peptide. *A*, Extracted ion chromatogram, *m/z* 891.9981 (+4). *t*_R_ 35.30 min. *B*, observed peaks at *m/z* 891.9981 (+4) and 1188.9969 (+3).**


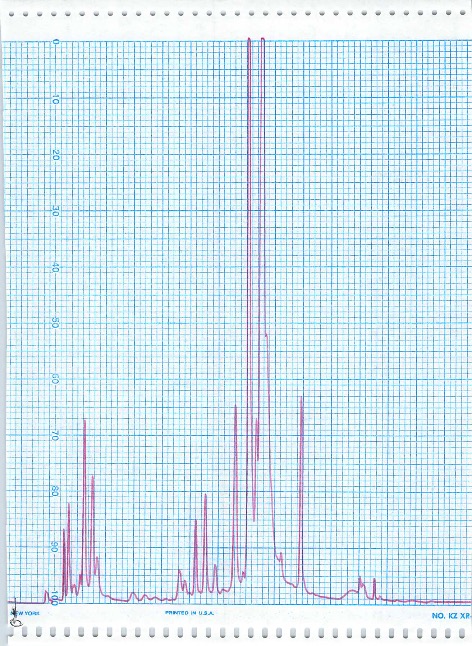


**Figure S4. HPLC purification of *N*^2^-cystamine-dG-modified oligonucleotide.** The oligonucleotide of interest eluted at 16.5 min (marked with arrow).

**Figure S5.** **MALDI spectrum of *N*^2^-cystamine-dG-modified oligonucleotide.** Expected [MH]^+^ 5890.0147, found [MH]^+^ 5891.6021.


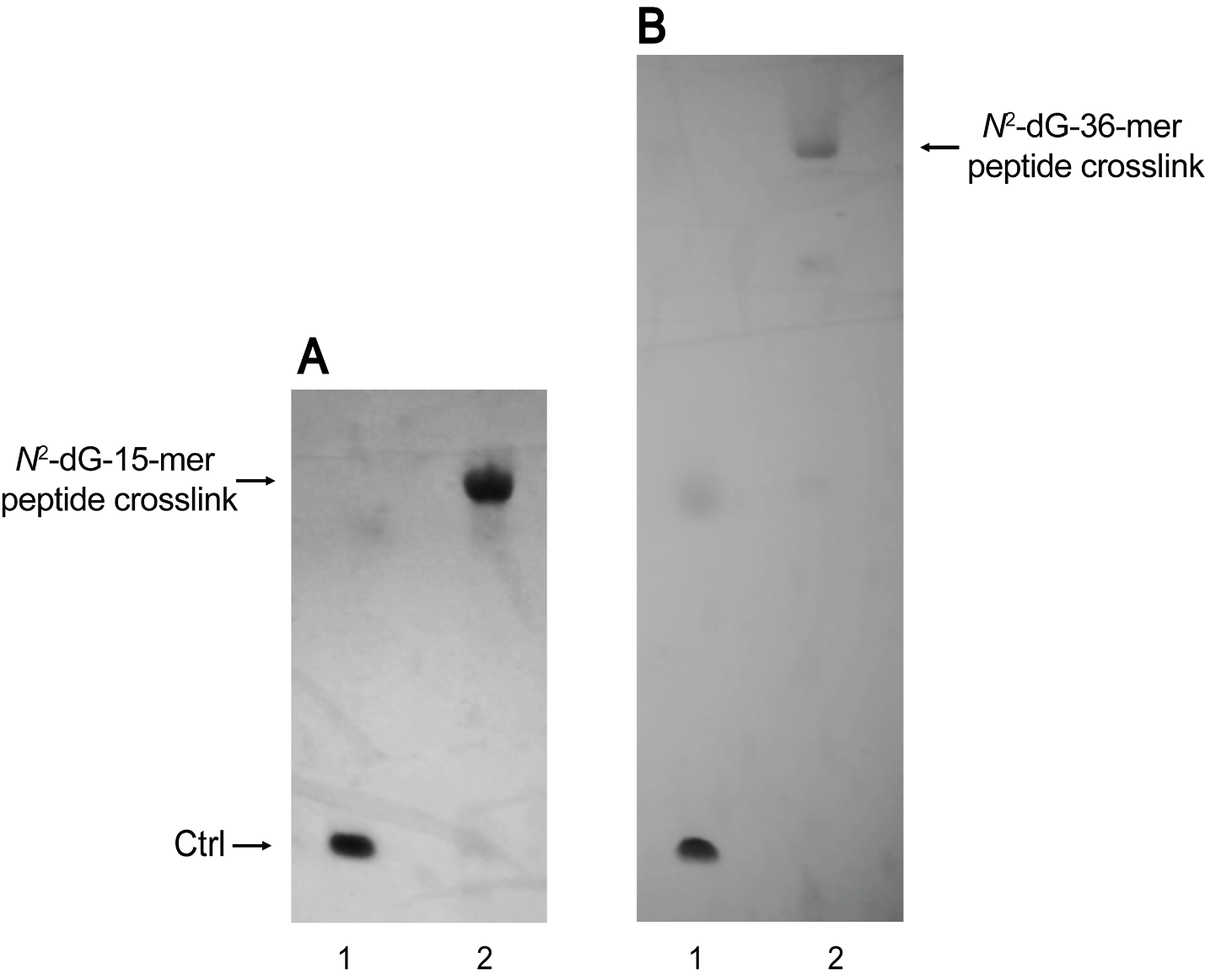


**Figure S6.** **Gel purification (PAGE, 20%, 7 M urea) of *N*^2^-dG-15-mer (*A*) and 36-mer (*B*) peptide crosslinks.** Lane 1*A* and *B*: 19-mer DNA template. Lanes 2*A* and 2*B*: *N*^2^-dG-15-mer and 36-mer peptide crosslink reaction mixtures.


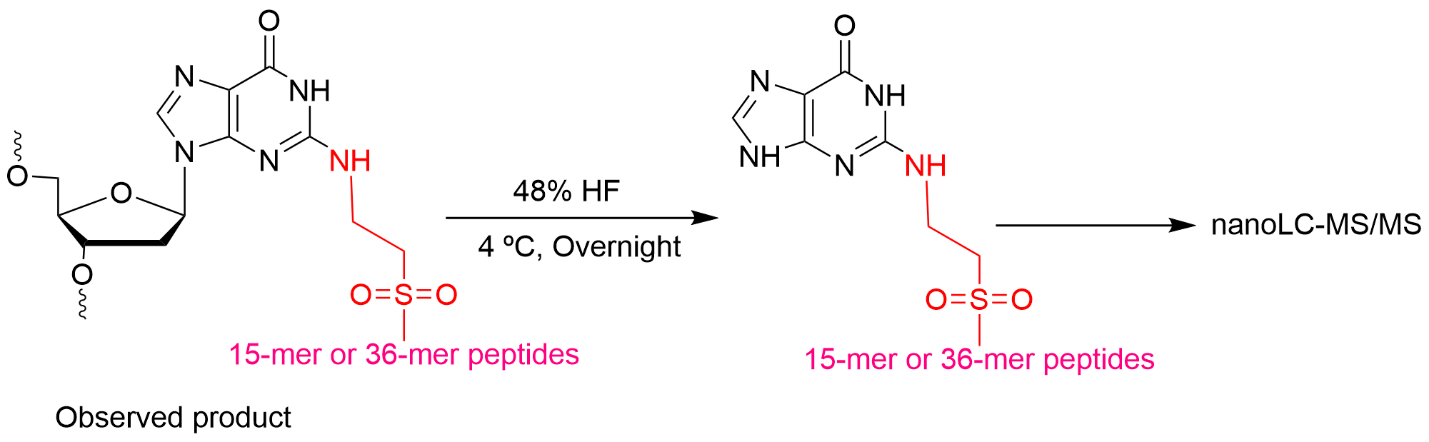


**Figure S7. HF treatment and nano-LC-ESI-MS/MS of *N*^2^-dG DNA-peptide crosslinks.**


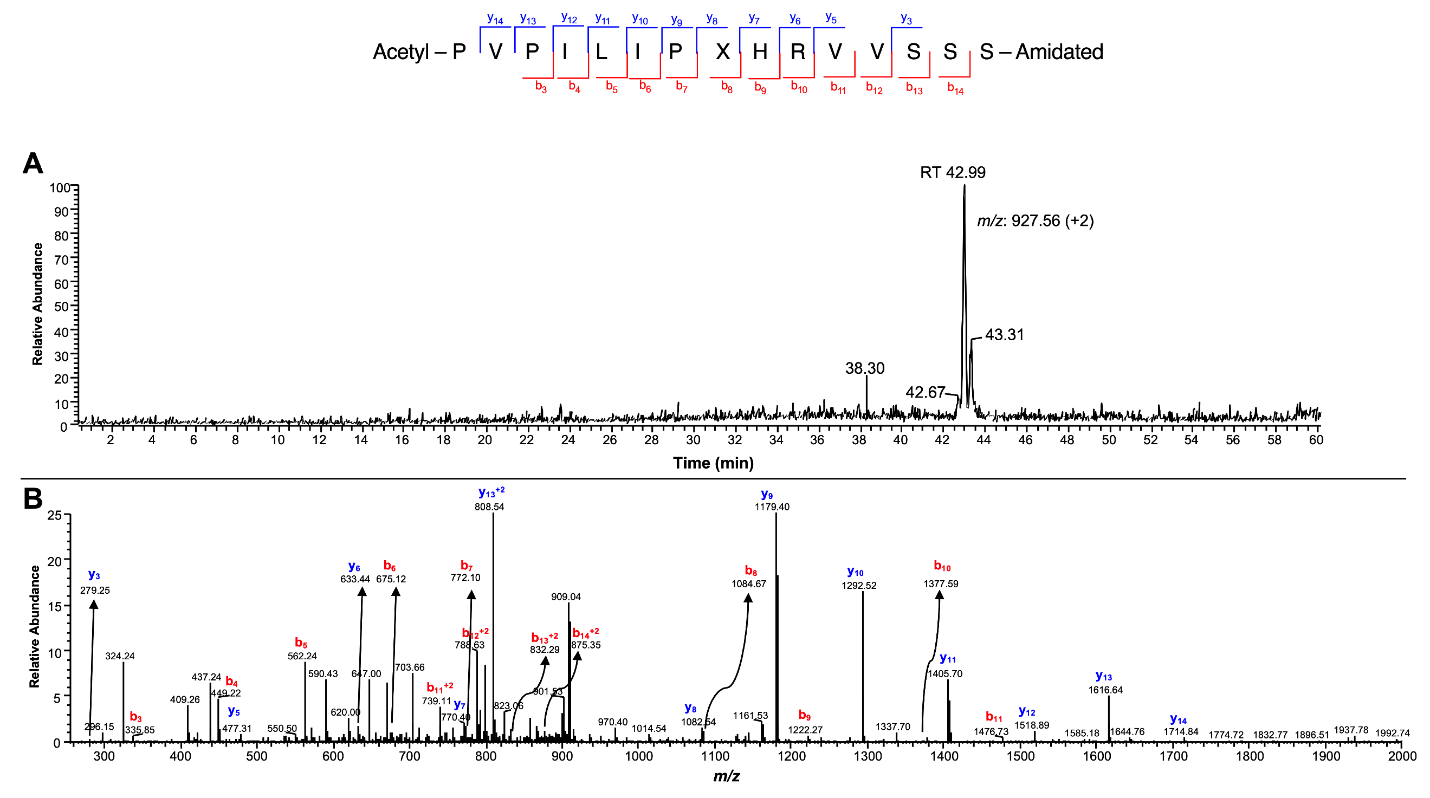


**Figure S8. LC-ESI-MS/MS analysis of HF-treated *N*^2^-dG-15-mer peptide crosslink.** *A*, extracted ion chromatogram for *m/z* 927.56 (+2, *t*_R_ 42.99 min). *B*, CID spectrum for *m/z* 927.56 (+2). See Table S2 for fragment assignments. b-ions are in red and y-ions in blue. X, cysteine crosslinked to dG (oxidized form).

**Table S2**

**Observed and theoretical CID fragments of *m/z* 927.56 (+2) for HF-treated *N*^2^-dG-15-mer peptide crosslink**

| Fragment assignment | *m/z* theoretical | *m/z* observed |
| --- | --- | --- |
| Acetyl-PVP (b_3_, +1) | 336.19 | 335.85 |
| Acetyl-PVPI (b_4_, +1) | 449.27 | 449.22 |
| Acetyl-PVPIL (b_5_, +1) | 562.36 | 562.24 |
| Acetyl-PVPILI (b_6_, +1) | 675.44 | 675.12 |
| Acetyl-PVPILIP (b_7_, +1) | 772.49 | 772.10 |
| Acetyl-PVPILIPX (b_8_, +1) | 1084.68 | 1084.67 |
| Acetyl-PVPILIPXH (b_9_, +1) | 1221.74 | 1222.27 |
| Acetyl-PVPILIPXHRV (b_10_, +1) | 1377.84 | 1377.59 |
| Acetyl-PVPILIPXHRVV (b_11_, +2) | 738.96 | 739.11 |
| Acetyl-PVPILIPXHRVV (b_12_, +2) | 788.49 | 788.63 |
| Acetyl-PVPILIPXHRVVS (b_13_, +2) | 832.01 | 832.29 |
| Acetyl-PVPILIPXHRVVSS (b_14_, +2) | 875.52 | 875.35 |
| VPILIPXHRVVSSS-Amidated (y_14_, +1) | 1715.04 | 1714.84 |
| PILIPXHRVVSSS-Amidated (y_13_, +2) | 808.49 | 808.54 |
| ILIPXHRVVSSS-Amidated (y_12_, +1) | 1518.92 | 1518.89 |
| LIPXHRVVSSS-Amidated (y_11_, +1) | 1405.83 | 1405.70 |
| IPXHRVVSSS-Amidated (y_10_, +1) | 1292.75 | 1292.52 |
| PXHRVVSSS-Amidated (y_9_, +1) | 1179.67 | 1179.40 |
| XHRVVSSS-Amidated (y_8_, +1) | 1082.61 | 1082.54 |
| HRVVSSS-Amidated (y_7_, +1) | 770.43 | 770.40 |
| RVVSSS-Amidated (y_6_, +1) | 633.37 | 633.44 |
| VVSSS-Amidated (y_5_, +1) | 477.26 | 477.31 |
| SSS-Amidated (y_3_, +1) | 279.13 | 279.25 |

X, cysteine crosslinked to dG (oxidized form)


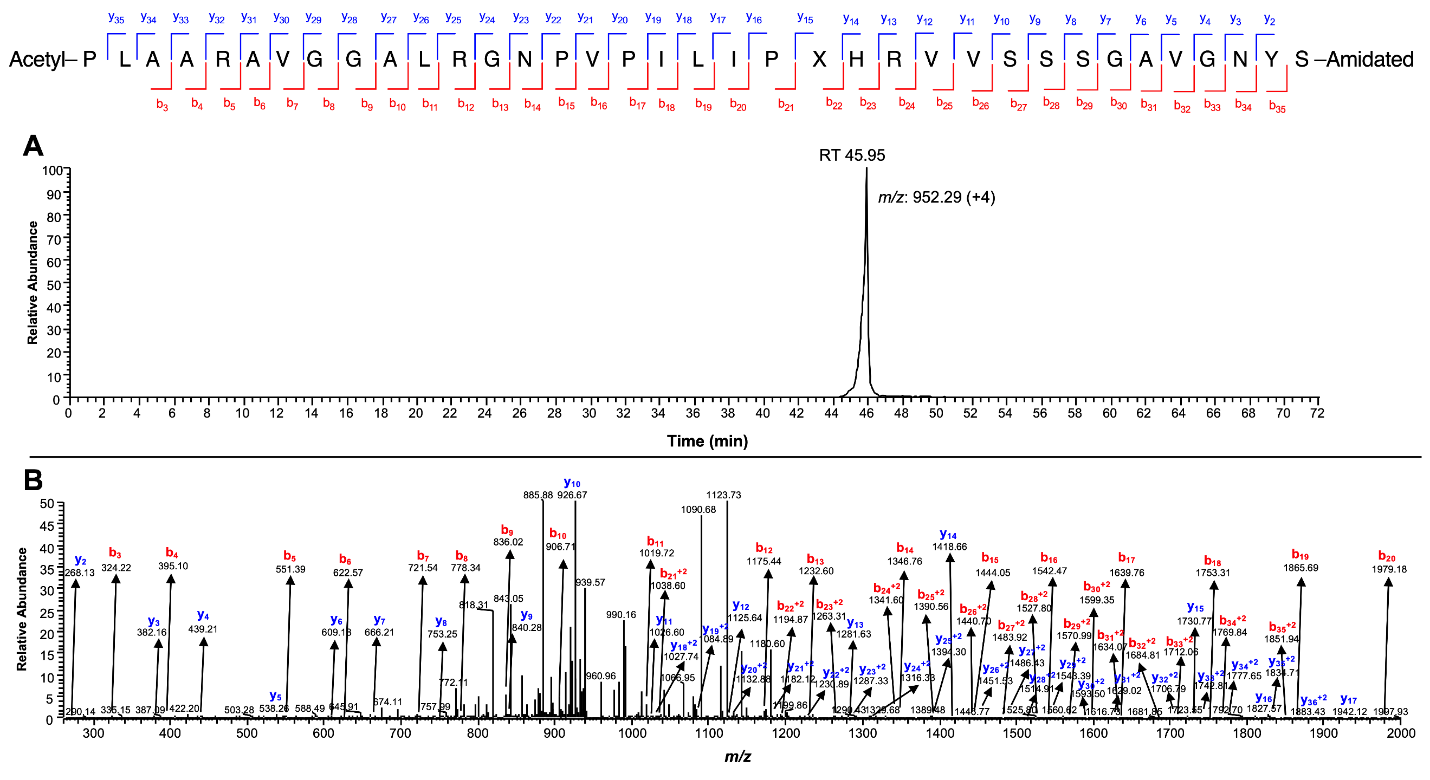


**Figure S9. LC-ESI-MS/MS analysis of HF-treated *N*^2^-dG-36-mer peptide crosslink.** *A*, Extracted ion chromatogram for *m/z* 952.29 (+4, *t*_R_ 45.95 min). *B*, CID spectrum for *m/z* 952.29 (+4). See Table S3 for fragment assignments. b-ions are in red and y-ions represented are in blue. X, cysteine crosslinked to dG (oxidized form).

**Table S3**

**Observed and theoretical CID fragments of *m/z* 952.29 (+4) for HF-treated *N*^2^-dG-36-mer peptide crosslink**

| Fragment assignment | *m/z* theoretical | *m/z* observed |
| --- | --- | --- |
| Acetyl-PLA (b_3_, +1) | 324.19 | 324.22 |
| Acetyl-PLAA (b_4_, +1) | 395.22 | 395.10 |
| Acetyl-PLAAR (b_5_, +1) | 551.33 | 551.39 |
| Acetyl-PLAARA (b_6_, +1) | 622.36 | 622.57 |
| Acetyl-PLAARAV (b_7_, +1) | 721.43 | 721.54 |
| Acetyl-PLAARAVG (b_8_, +1) | 778.45 | 778.34 |
| Acetyl-PLAARAVGG (b_9_, +1) | 835.47 | 836.02 |
| Acetyl-PLAARAVGGA (b_10_, +1) | 906.51 | 906.71 |
| Acetyl-PLAARAVGGAL (b_11_, +1) | 1019.59 | 1019.72 |
| Acetyl-PLAARAVGGALR (b_12_, +1) | 1175.70 | 1175.44 |
| Acetyl-PLAARAVGGALRG (b_13_, +1) | 1232.72 | 1232.60 |
| Acetyl-PLAARAVGGALRGN (b_14_, +1) | 1346.76 | 1346.76 |
| Acetyl-PLAARAVGGALRGNP (b_15_, +1) | 1443.81 | 1444.05 |
| Acetyl-PLAARAVGGALRGNPV (b_16_, +1) | 1542.88 | 1542.47 |
| Acetyl-PLAARAVGGALRGNPVP (b_17_, +1) | 1639.93 | 1639.76 |
| Acetyl-PLAARAVGGALRGNPVPI (b_18_, +1) | 1753.02 | 1753.31 |
| Acetyl-PLAARAVGGALRGNPVPIL (b_19_, +1) | 1866.10 | 1865.69 |
| Acetyl-PLAARAVGGALRGNPVPILI (b_20_, +1) | 1979.19 | 1979.18 |
| Acetyl-PLAARAVGGALRGNPVPILIP (b_21_, +2) | 1038.62 | 1038.60 |
| Acetyl-PLAARAVGGALRGNPVPILIPX (b_22_, +2) | 1194.72 | 1194.87 |
| Acetyl-PLAARAVGGALRGNPVPILIPXH (b_23_, +2) | 1263.25 | 1263.31 |
| Acetyl-PLAARAVGGALRGNPVPILIPXHR (b_24_, +2) | 1341.30 | 1341.60 |
| Acetyl-PLAARAVGGALRGNPVPILIPXHRV (b_25_, +2) | 1390.83 | 1390.56 |
| Acetyl-PLAARAVGGALRGNPVPILIPXHRVV (b_26_, +2) | 1440.37 | 1440.70 |
| Acetyl-PLAARAVGGALRGNPVPILIPXHRVVS (b_27_, +2) | 1483.88 | 1483.92 |
| Acetyl-PLAARAVGGALRGNPVPILIPXHRVVSS (b_28_, +2) | 1527.40 | 1527.80 |
| Acetyl-PLAARAVGGALRGNPVPILIPXHRVVSSS (b_29_, +2) | 1570.91 | 1570.99 |
| Acetyl-PLAARAVGGALRGNPVPILIPXHRVVSSSG (b_30_, +2) | 1599.42 | 1599.35 |
| Acetyl-PLAARAVGGALRGNPVPILIPXHRVVSSSGA (b_31_, +2) | 1634.94 | 1634.07 |
| Acetyl-PLAARAVGGALRGNPVPILIPXHRVVSSSGAV (b_32_, +2) | 1684.48 | 1684.81 |
| Acetyl-PLAARAVGGALRGNPVPILIPXHRVVSSSGAVG (b_33_, +2) | 1712.99 | 1712.06 |
| Acetyl-PLAARAVGGALRGNPVPILIPXHRVVSSSGAVGN (b_34_, +2) | 1770.01 | 1769.84 |
| Acetyl-PLAARAVGGALRGNPVPILIPXHRVVSSSGAVGNY (b_35_, +2) | 1851.54 | 1851.94 |

| Fragment assignment | *m/z* theoretical | *m/z* observed |
| --- | --- | --- |
| Acetyl-PLAARAVGGALRGNPVPILIPXHRVVSSSGAVGNYS-Amidated (y_36_, +2) | 1882.56 | 1883.43 |
| LAARAVGGALRGNPVPILIPXHRVVSSSGAVGNYS-Amidated (y_35_, +2) | 1834.04 | 1834.71 |
| AARAVGGALRGNPVPILIPXHRVVSSSGAVGNYS-Amidated (y_34_, +2) | 1777.50 | 1777.65 |
| ARAVGGALRGNPVPILIPXHRVVSSSGAVGNYS-Amidated (y_33_, +2) | 1741.98 | 1742.81 |
| RAVGGALRGNPVPILIPXHRVVSSSGAVGNYS-Amidated (y_32_, +2) | 1706.46 | 1706.79 |
| AVGGALRGNPVPILIPXHRVVSSSGAVGNYS-Amidated (y_31_, +2) | 1628.41 | 1629.02 |
| VGGALRGNPVPILIPXHRVVSSSGAVGNYS-Amidated (y_30_, +2) | 1592.89 | 1593.50 |
| GGALRGNPVPILIPXHRVVSSSGAVGNYS-Amidated (y_29_, +2) | 1543.36 | 1543.39 |
| GALRGNPVPILIPXHRVVSSSGAVGNYS-Amidated (y_28_, +2) | 1514.85 | 1514.91 |
| ALRGNPVPILIPXHRVVSSSGAVGNYS-Amidated (y_27_, +2) | 1486.33 | 1486.43 |
| LRGNPVPILIPXHRVVSSSGAVGNYS-Amidated (y_26_, +2) | 1450.82 | 1451.53 |
| RGNPVPILIPXHRVVSSSGAVGNYS-Amidated (y_25_, +2) | 1394.27 | 1394.30 |
| GNPVPILIPXHRVVSSSGAVGNYS-Amidated (y_24_, +2) | 1316.22 | 1316.33 |
| NPVPILIPXHRVVSSSGAVGNYS-Amidated (y_23_, +2) | 1287.71 | 1287.33 |
| PVPILIPXHRVVSSSGAVGNYS-Amidated (y_22_, +2) | 1230.69 | 1230.89 |
| VPILIPXHRVVSSSGAVGNYS-Amidated (y_21_, +2) | 1182.16 | 1182.12 |
| PILIPXHRVVSSSGAVGNYS-Amidated (y_20_, +2) | 1132.63 | 1132.88 |
| ILIPXHRVVSSSGAVGNYS-Amidated (y_19_, +2) | 1084.10 | 1084.89 |
| LIPXHRVVSSSGAVGNYS-Amidated (y_18_, +2) | 1027.56 | 1027.74 |
| IPXHRVVSSSGAVGNYS-Amidated (y_17_, +1) | 1941.04 | 1942.12 |
| PXHRVVSSSGAVGNYS-Amidated (y_16_, +1) | 1827.95 | 1827.57 |
| XHRVVSSSGAVGNYS-Amidated (y_15_, +1) | 1730.90 | 1730.77 |
| HRVVSSSGAVGNYS-Amidated (y_14_, +1) | 1418.71 | 1418.66 |
| RVVSSSGAVGNYS-Amidated (y_13_, +1) | 1281.65 | 1281.63 |
| VVSSSGAVGNYS-Amidated (y_12_, +1) | 1125.55 | 1125.64 |
| VSSSGAVGNYS-Amidated (y_11_, +2) | 1026.48 | 1026.60 |
| SSSGAVGNYS-Amidated (y_10_, +1) | 927.41 | 926.67 |
| SSGAVGNYS-Amidated (y_9_, +1) | 840.38 | 840.28 |
| SGAVGNYS-Amidated (y_8_, +1) | 753.35 | 753.25 |
| GAVGNYS-Amidated (y_7_, +1) | 666.32 | 666.21 |
| AVGNYS-Amidated (y_6_, +1) | 609.29 | 609.18 |
| VGNYS-Amidated (y_5_, +1) | 538.26 | 538.26 |
| GNYS-Amidated (y_4_, +1) | 439.19 | 439.21 |
| NYS-Amidated (y_3_, +1) | 382.17 | 382.16 |
| YS-Amidated (y_2_, +1) | 268.12 | 268.13 |

X, cysteine crosslinked to dG (oxidized form)


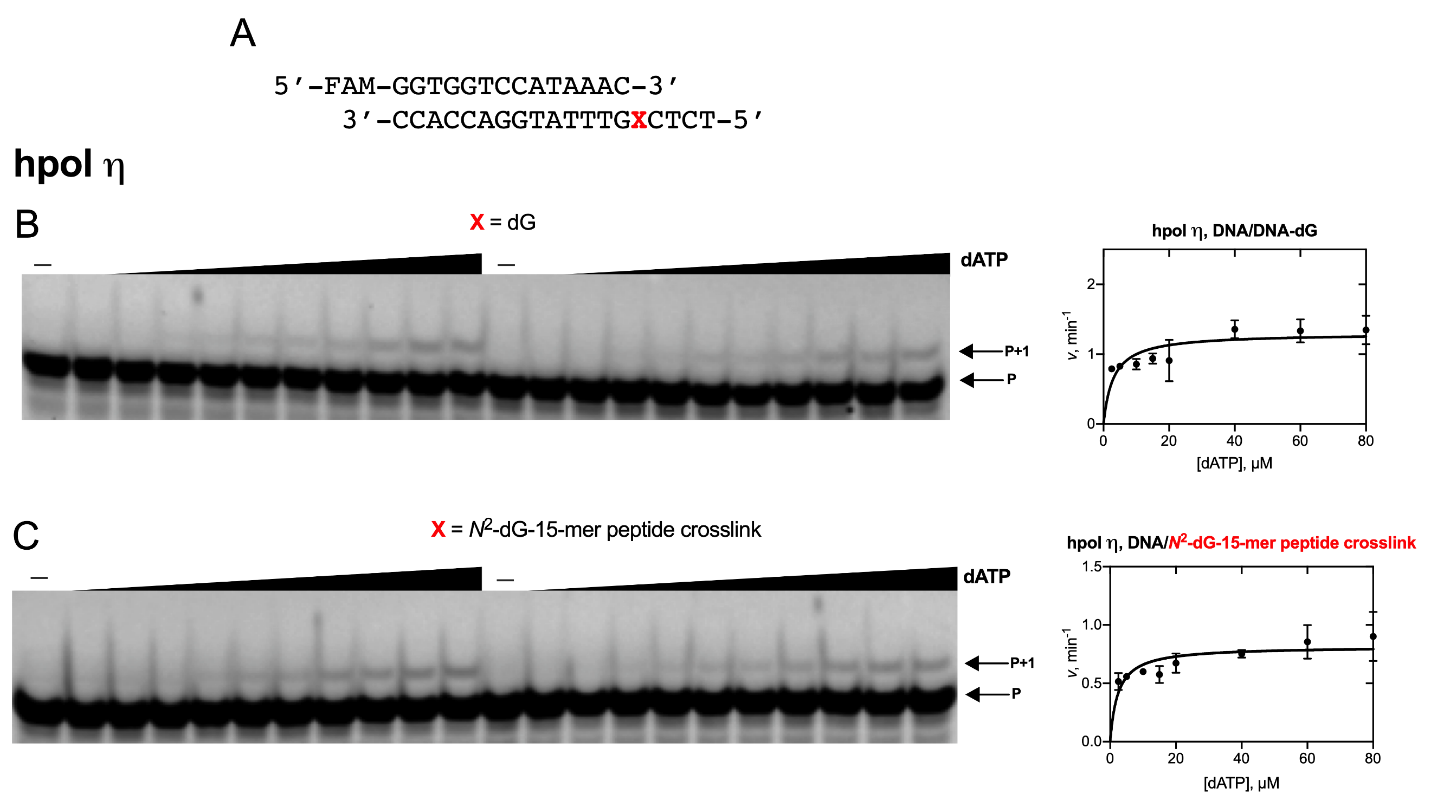


**Figure S10. Steady-state kinetic analysis of dATP insertion by hpol η.** *A*, 14-mer primer and template sequences, where X is dG or *N*^2^-dG-15-mer peptide crosslink. All reactions were done in duplicate at 37 °C for 5 min using hpol η: *B*, 1.2 nM; *C*, 1.8 nM. Varying concentrations of dATP were used (2.5-80 µM). Data points are shown as means ± SD (range); see Table 1 for *k*_cat_ and *K*_m_ values (estimated using fit to a hyperbolic equation in Prism) and Table S1 for the oligonucleotide sequences used. P, FAM-labeled 14-mer DNA primer.


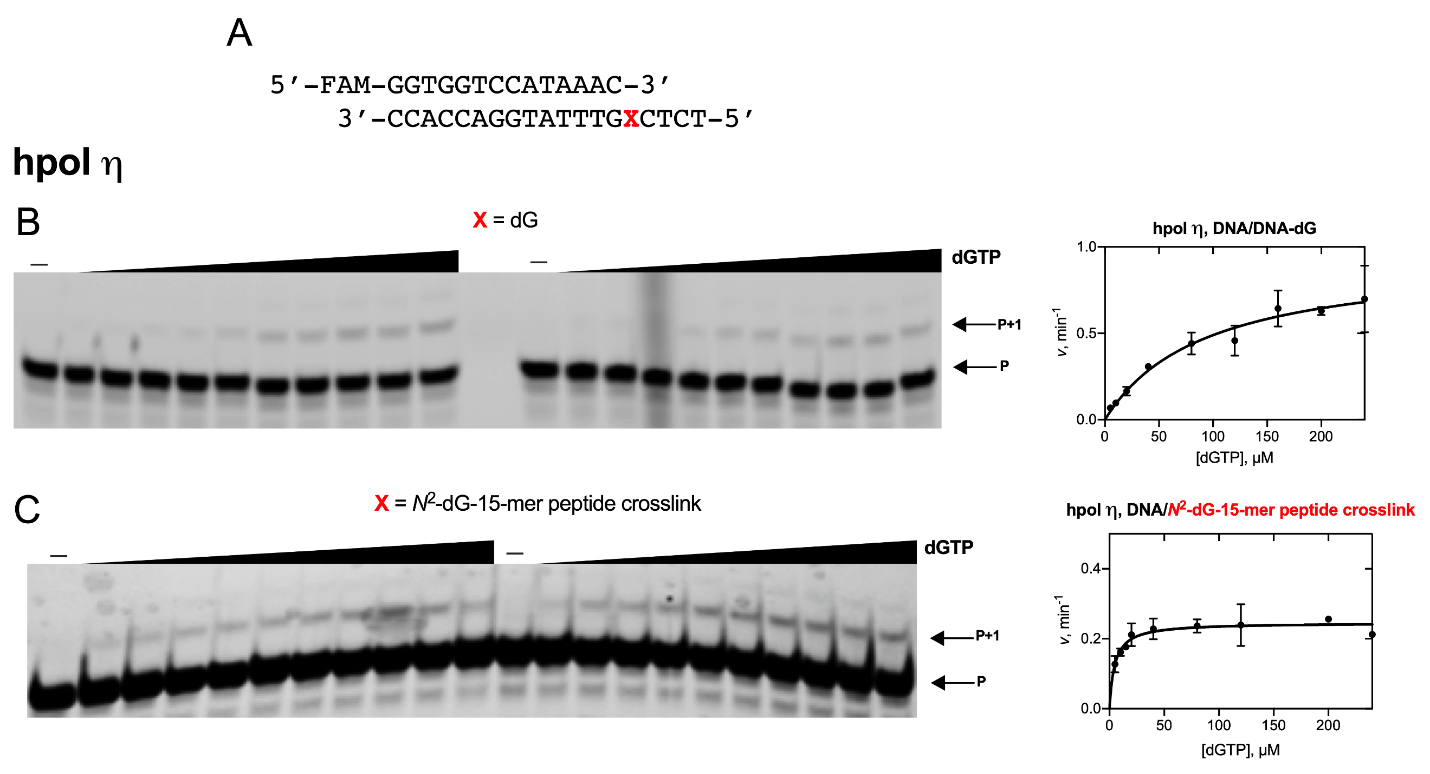


**Figure S11.** **Steady-state kinetic analysis of dGTP insertion by hpol η.** *A*, 14-mer primer and template sequences, where X is dG or *N*^2^-dG-15-mer peptide crosslink. All reactions were carried out in duplicate at 37 °C for 6 min using hpol η: 3 nM. Varying concentrations of dGTP were used 5-240 µM. Data points are shown as means ± SD (range); see Table 1 for *k*_cat_ and *K*_m_ values (fit to a hyperbolic equation in Prism) and Table S1 for the oligonucleotide sequences used. P, FAM-labeled 14-mer DNA primer.


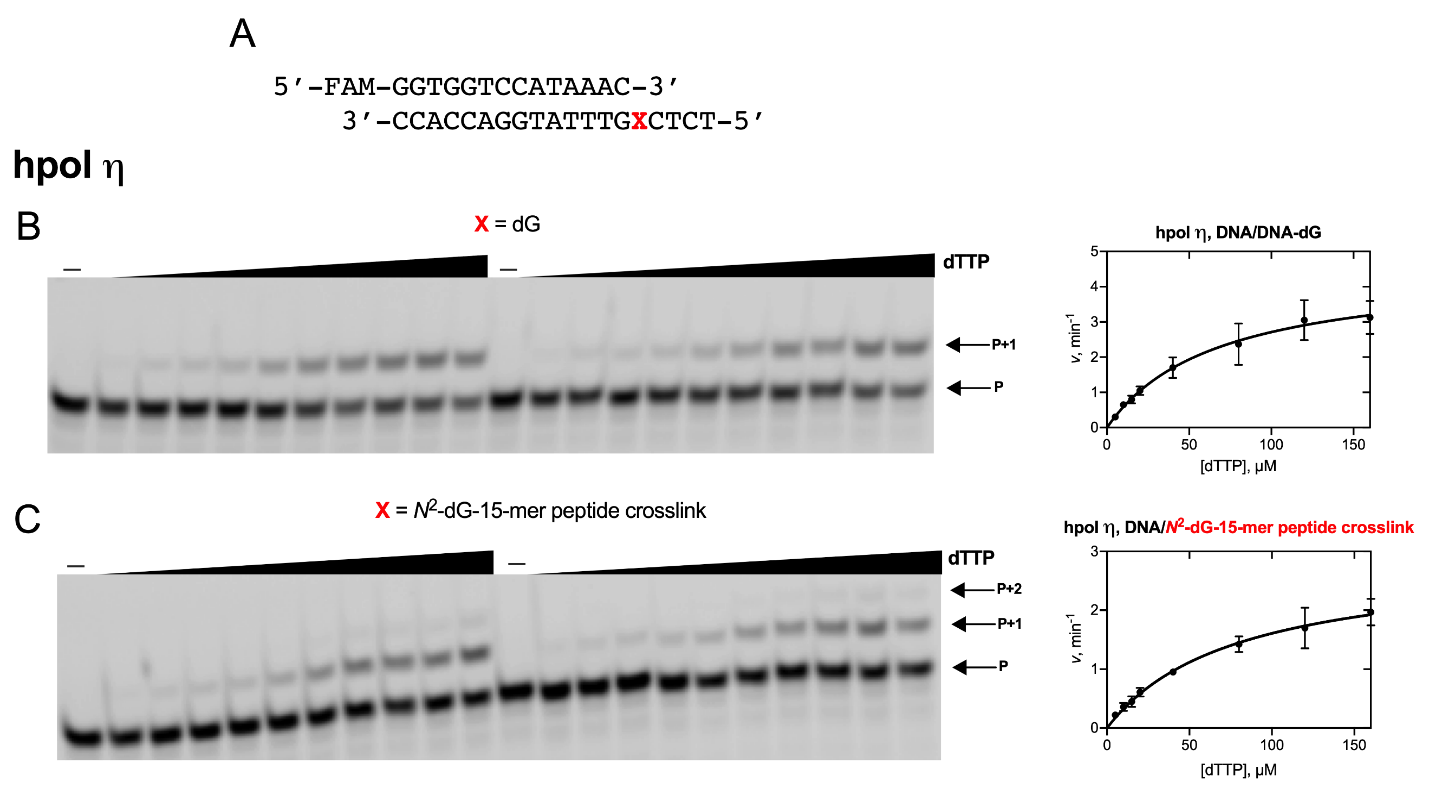


**Figure S12.** **Steady-state kinetic analysis of dTTP insertion by hpol η.** *A*, 14-mer primer and template sequences, where X is dG or *N*^2^-dG-15-mer peptide crosslink. All reactions were carried out in duplicate at 37 °C for 6 min using hpol η: *B*, 2.1 nM; *C*, 2.8 nM. Varying concentrations of dTTP were used (5-160 µM). Data points are shown as means ± SD (range); see Table 1 for *k*_cat_ and *K*_m_ values (fit to a hyperbolic equation in Prism) and Table S1 for the oligonucleotide sequences used. P, FAM-labeled 14-mer DNA primer.

**Figure S13.** **PAGE (20%, 7 M urea) of full-length extension reactions employing hpol η (0.75 µM) in the presence of a mixture of dNTPs.** *A*, primer-template complex; *B*, full-length extension reactions for unmodified and *N*^2^-dG-15-mer peptide crosslink template-primer complex (Lanes 1 and 4 included FAM-labeled dU-containing 14-mer primer, Lanes 2 and 5 included fully-extended products, and Lanes 3 and 6 included cleaved FAM-labeled primer after UDG and piperidine treatment. All reactions were carried out at 37 °C for 4 h.


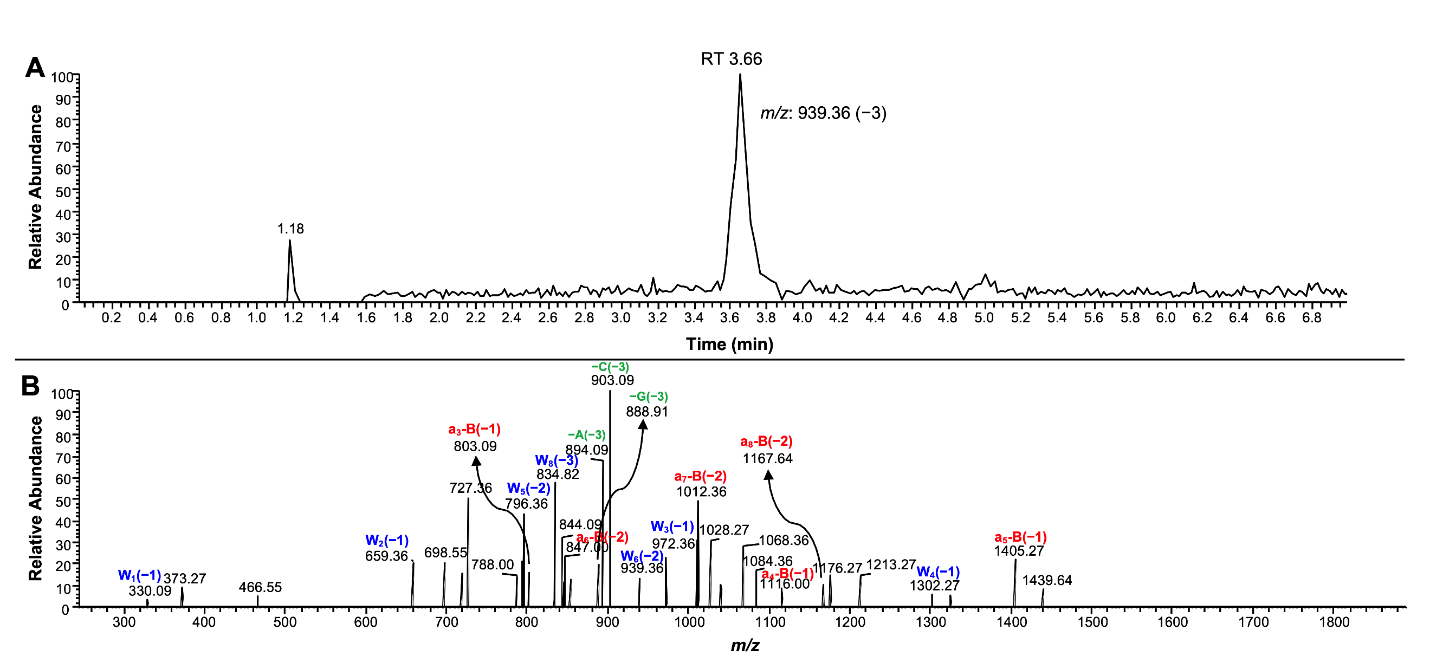


**Figure S14.** **LC-ESI-MS/MS sequencing analysis of full-length extension reactions for control template by hpol η in the presence of dNTPs.** Extracted ion chromatogram and CID spectrum of *m/z* 939.36 (−3, *t*_R_ 3.66 min) for control DNA.

**Table S4**

**Observed and theoretical CID fragments of *m/z* 939.36 (−3) from full-length extended products for control template**

The extended product sequence is 5′-pAAAC**C**GAGA-3′ (Fig. 14B), indicating the insertion of C.

| **Fragment assignment** | ***m/z* observed** | ***m/z* theoretical** |
| --- | --- | --- |
| 5′-pAAA (a_3_-B, −1) | 803.09 | 803.49 |
| 5′-pAAAC (a_4_-B, −1) | 1116.64 | 1116.70 |
| 5′-pAAACC (a_5_-B, −1) | 1405.27 | 1405.89 |
| 5′-pAAACCG (a_6_-B, −2) | 847.00 | 847.03 |
| 5′-pAAACCGA (a_7_-B, −2) | 1012.36 | 1011.63 |
| 5′-pAAACCGAG (a_8_-B, −2) | 1167.64 | 1168.24 |
| pAACCGAGA-3′ (W_8_, −3) | 834.82 | 834.87 |
| pCCGAGA-3′ (W_6_, −2) | 939.36 | 939.60 |
| pCGAGA-3′ (W_5_, −2) | 796.36 | 795.01 |
| pAGA-3′ (W_3_, −1) | 972.36 | 972.63 |
| pGA-3′ (W_2_, −1) | 659.36 | 659.42 |
| pA-3′ (W_1_, −1) | 330.09 | 330.21 |


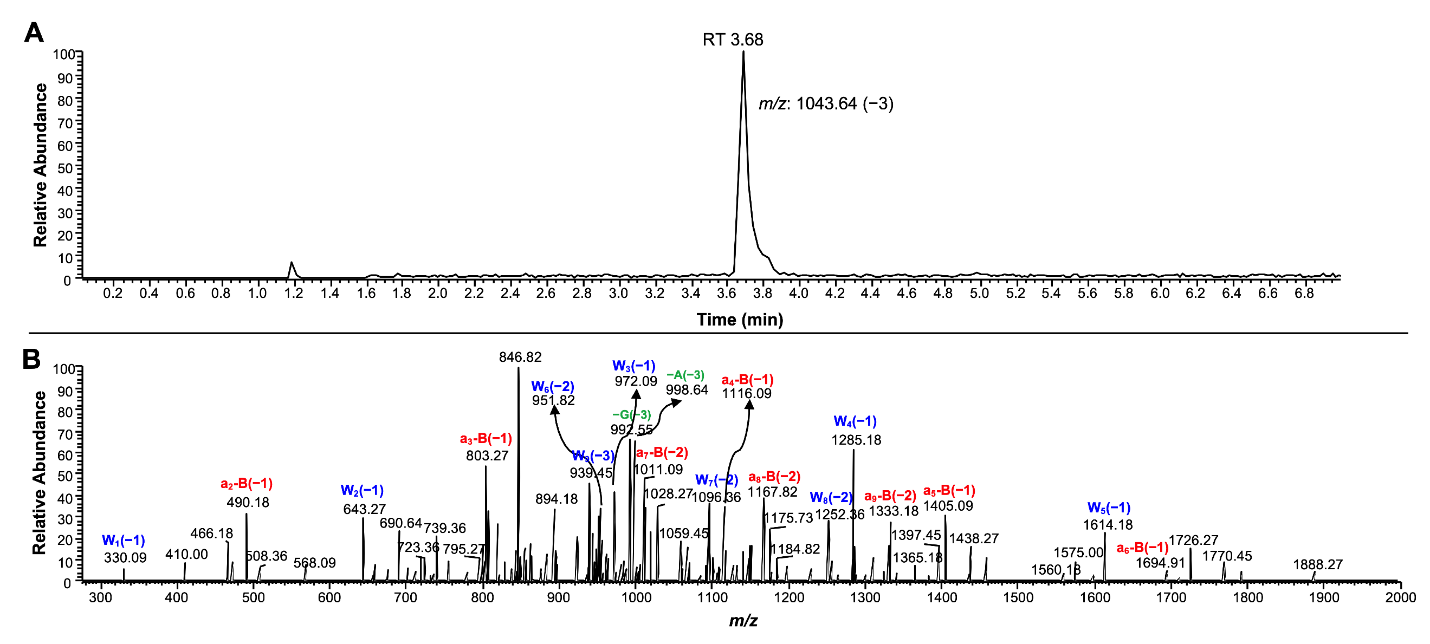


**Figure S15. LC-ESI-MS/MS sequence analysis of full-length extension reactions for control template by hpol η in the presence of dNTPs.** *A*, extracted ion chromatogram for *m/z* 1043.64 (−3, *t*_R_ 3.68 min); *B*, CID spectrum of *m/z* 1043.64 (−3). See Table S5 for fragment assignments.

**Table S5**

**Observed and theoretical CID fragments of *m/z* 1043.64 (−3) from full-length extended products for control template**

The extended product sequence is 5′-pAAAC**C**GAGAA-3′ (Fig. S15*B*), indicating insertion of C with blunt end addition of A.

| **Fragment assignment** | ***m/z* observed** | ***m/z* theoretical** |
| --- | --- | --- |
| 5′-pAA (a_2_-B, −1) | 490.18 | 490.28 |
| 5′-pAAA (a_3_-B, −1) | 803.27 | 803.49 |
| 5′-pAAAC (a_4_-B, −1) | 1116.09 | 1116.70 |
| 5′-pAAACC (a_5_-B, −1) | 1405.09 | 1405.89 |
| 5′-pAAACCG (a_6_-B, −1) | 1694.91 | 1695.07 |
| 5′-pAAACCGA (a_7_-B, −2) | 1011.09 | 1011.63 |
| 5′-pAAACCGAG (a_8_-B, −2) | 1167.82 | 1168.24 |
| 5′-pAAACCGAGA (a_9_-B, −2) | 1333.18 | 1332.84 |
| pAACCGAGAA-3′ (W_9_, −3) | 939.45 | 939.27 |
| pACCGAGAA-3′ (W_8_, −2) | 1252.36 | 1252.81 |
| pCCGAGAA-3′ (W_7_, −2) | 1096.36 | 1096.20 |
| pCGAGAA-3′ (W_6_, −2) | 951.82 | 951.61 |
| pGAGAA-3′ (W_5_, −1) | 1614.18 | 1615.05 |
| pAGAA-3′ (W_4_, −1) | 1285.18 | 1285.84 |
| pGAA-3′ (W_3_, −1) | 972.09 | 972.63 |
| pAA-3′ (W_2_, −1) | 643.27 | 643.42 |
| pA-3′ (W_1_, −1) | 330.09 | 330.21 |


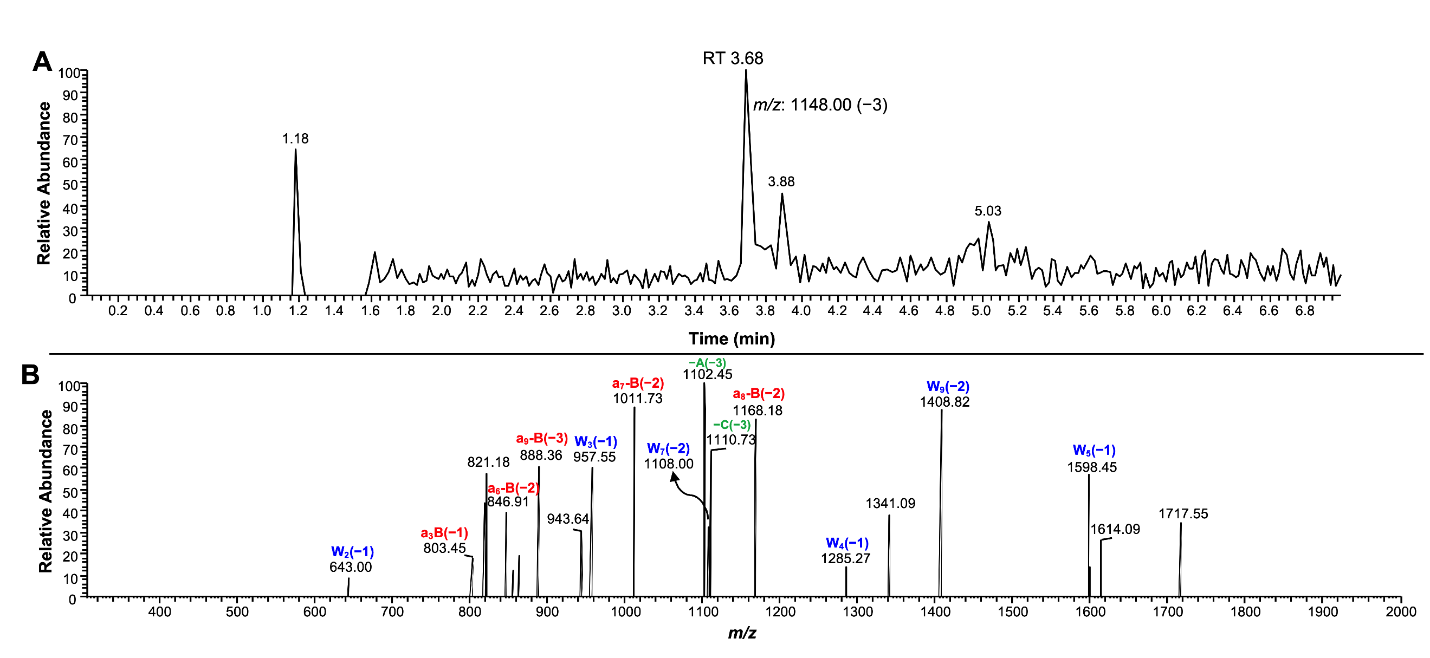


**Figure S16. LC-ESI-MS/MS sequencing analysis of full-length extension reactions for control template by hpol η in the presence of dNTPs.** *A*, extracted ion chromatogram for *m/z* 1148.00 (−3, *t*_R_ 3.68 min); *B*, CID spectrum of *m/z* 1148.00 (−3). See Table S6 for fragment assignment.

**Table S6**

**Observed and theoretical CID fragments of *m/z* 1148.00 (−3) from full-length extended products for control template**

The extended product sequence is 5′-pAAAC**C**GAGAAA-3′ (Fig. S16*B*), indicating insertion of C with blunt end addition of AA.

| **Fragment assignment** | ***m/z* observed** | ***m/z* theoretical** |
| --- | --- | --- |
| 5′-pAAA (a_3_-B, −1) | 803.45 | 803.49 |
| 5′-pAAACCG (a_6_-B, −2) | 846.91 | 847.03 |
| 5′-pAAACCGA (a_7_-B, −2) | 1011.73 | 1011.63 |
| 5′-pAAACCGAG (a_8_-B, −2) | 1168.18 | 1168.24 |
| 5′-pAAACCGAGA (a_9_-B, −3) | 888.36 | 888.23 |
| pACCGAGAAA-3′ (W_9_, −2) | 1408.82 | 1409.41 |
| pCGAGAAA-3′ (W_7_, −2) | 1108.00 | 1108.22 |
| pAGAAA-3′ (W_5_, −1) | 1598.45 | 1599.05 |
| pGAAA-3′ (W_4_, −1) | 1285.27 | 1285.84 |
| pAAA-3′ (W_3_, −1) | 957.55 | 956.63 |
| pAA-3′ (W_2_, −1) | 643.00 | 643.42 |

**Table S7**

**Observed and theoretical CID fragments of *m/z* 939.36 (−3) from full-length extended products for *N*^2^-dG-15-mer peptide crosslink**

The extended product sequence is 5′-pAAAC**C**GAGA-3′ (Fig. 8), indicating the insertion of C.

| **Fragment assignment** | ***m/z* observed** | ***m/z* theoretical** |
| --- | --- | --- |
| 5′-pAA (a_2_-B, −1) | 490.18 | 490.28 |
| 5′-pAAA (a_3_-B, −1) | 803.09 | 803.49 |
| 5′-pAAACCGA (a_7_-B, −2) | 1011.82 | 1011.63 |
| 5′-pAAACCGAG (a_8_-B, −2) | 1169.55 | 1168.24 |
| pACCGAGA-3′ (W_7_, −2) | 1096.82 | 1096.20 |
| pCCGAGA-3′ (W_6_, −2) | 940.18 | 939.60 |
| pAGA-3′ (W_3_, −1) | 972.27 | 972.63 |
| pGA-3′ (W_2_, −1) | 659.09 | 659.42 |
| pA-3′ (W_1_, −1) | 330.27 | 330.21 |


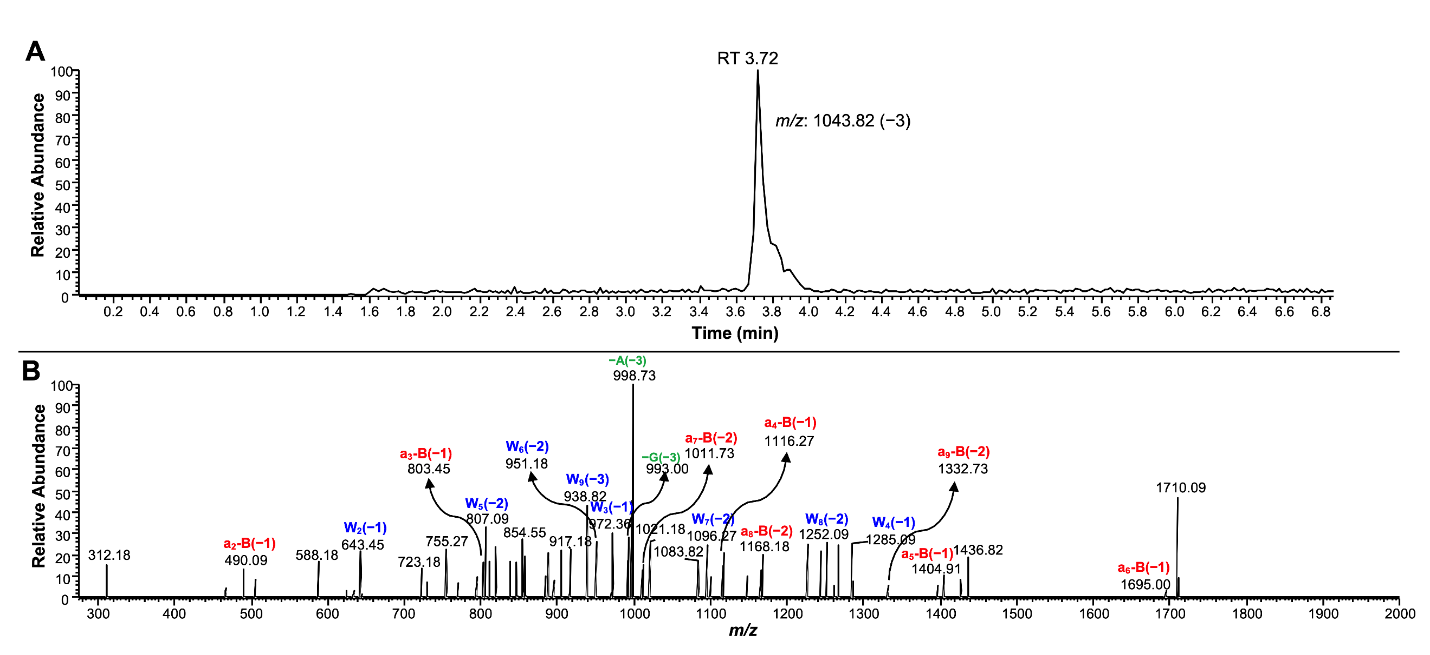


**Figure S17.** **LC-ESI-MS/MS sequence analysis of full-length extension reactions for *N*^2^-dG-15-mer peptide crosslink by hpol η in the presence of dNTPs.** *A*, extracted ion chromatogram for *m/z* 1043.82 (−3, *t*_R_ 3.72 min); *B*, CID spectrum of *m/z* 1043.82 (−3). See Table S8 for fragment assignments.

**Table S8**

**Observed and theoretical CID fragments of *m/z* 1043.82 (−3) from full-length extended products for *N*^2^-dG-15-mer peptide crosslink**

The extended product sequence is 5′-pAAAC**C**GAGAA-3′ (Fig. S17*B*), indicating insertion of C with blunt end addition of A.

| **Fragment assignment** | ***m/z* observed** | ***m/z* theoretical** |
| --- | --- | --- |
| 5′-pAA (a_2_-B, −1) | 490.09 | 490.28 |
| 5′-pAAA (a_3_-B, −1) | 803.45 | 803.49 |
| 5′-pAAAC (a_4_-B, −1) | 1116.27 | 1116.70 |
| 5′-pAAACC (a_5_-B, −1) | 1404.91 | 1405.89 |
| 5′-pAAACCG (a_6_-B, −1) | 1695.00 | 1695.07 |
| 5′-pAAACCGA (a_7_-B, −2) | 1011.73 | 1011.63 |
| 5′-pAAACCGAG (a_8_-B, −2) | 1168.18 | 1168.24 |
| 5′-pAAACCGAGA (a_9_-B, −2) | 1332.73 | 1332.84 |
| pAACCGAGAA-3′ (W_9_, −3) | 938.82 | 939.27 |
| pACCGAGAA-3′ (W_8_, −2) | 1252.09 | 1252.81 |
| pCCGAGAA-3′ (W_7_, −2) | 1096.27 | 1096.20 |
| pCGAGAA-3′ (W_6_, −2) | 951.18 | 951.61 |
| pGAGAA-3′ (W_5_, −2) | 807.09 | 807.02 |
| pAGAA-3′ (W_4_, −1) | 1285.09 | 1285.84 |
| pGAA-3′ (W_3_, −1) | 972.36 | 972.63 |
| pAA-3′ (W_2_, −1) | 643.45 | 643.42 |


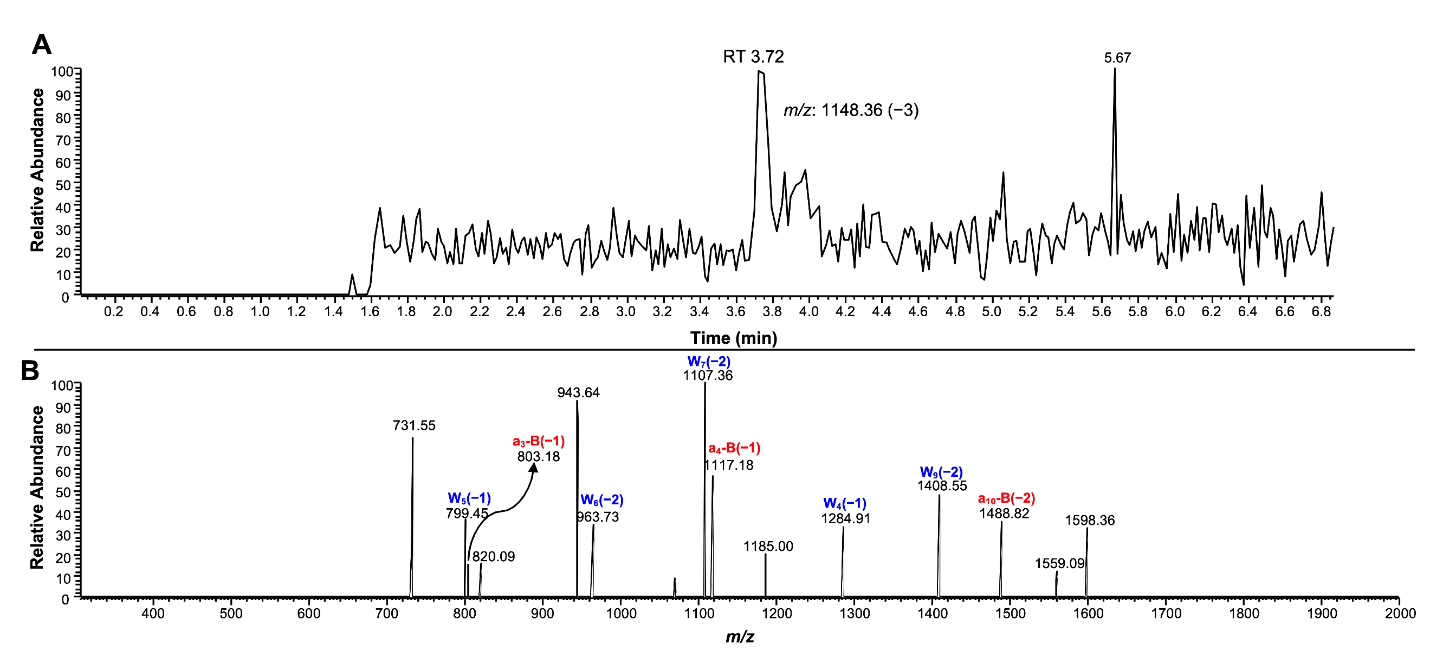


**Figure S18. LC-ESI-MS/MS sequencing analysis of full-length extension reactions for *N*^2^-dG-15-mer peptide crosslink by hpol η in the presence of dNTPs.** *A*, extracted ion chromatogram for *m/z* 1148.36 (−3, *t*_R_ 3.72 min); *B*, CID spectrum of *m/z* 1148.36 (−3). See Table S9 for fragment assignment.

**Table S9**

**Observed and theoretical CID fragments of *m/z* 1148.36 (−3) from full-length extended products for *N*^2^-dG-15-mer peptide crosslink**

The extended product sequence is 5′-pAAAC**C**GAGAAA-3′ (Fig. S18*B*), indicating insertion of C with blunt end addition of AA.

| **Fragment assignment** | ***m/z* observed** | ***m/z* theoretical** |
| --- | --- | --- |
| 5′-pAAA (a_3_-B, −1) | 803.18 | 803.49 |
| 5′-pAAAC (a_4_-B, −1) | 1117.18 | 1116.70 |
| 5′-pAAACCGAGAA (a_10_-B, −2) | 1488.82 | 1489.45 |
| pACCGAGAAA-3′ (W_9_, −2) | 1408.55 | 1409.41 |
| pCGAGAAA-3′ (W_7_, −2) | 1107.36 | 1108.22 |
| pGAGAAA-3′ (W_6_, −2) | 963.73 | 963.62 |
| pAGAAA-3′ (W_5_, −2) | 799.45 | 799.02 |
| pGAAA-3′ (W_4_, −1) | 1284.91 | 1285.84 |

**Reference**

1. Ghodke, P. P., Gonzalez-Vasquez, G., Wang, H., Johnson, K. M., Sedgeman, C. A., and Guengerich, F. P. (2021) Enzymatic bypass of an *N*^6^-deoxyadenosine DNA–ethylene dibromide–peptide cross-link by translesion DNA polymerases. *J. Biol. Chem.* **296**, 100444
